# The Historical Evolution and Significance of Multiple Sequence Alignment in Molecular Structure and Function Prediction

**DOI:** 10.3390/biom14121531

**Published:** 2024-11-29

**Authors:** Chenyue Zhang, Qinxin Wang, Yiyang Li, Anqi Teng, Gang Hu, Qiqige Wuyun, Wei Zheng

**Affiliations:** 1 NITFID, School of Statistics and Data Science, LPMC and KLMDASR, Nankai University, Tianjin 300071, China; 1120220056@mail.nankai.edu.cn (C.Z.); 2120220137@mail.nankai.edu.cn (Y.L.); huggs@nankai.edu.cn (G.H.); 2 Suzhou New & High-Tech Innovation Service Center, Suzhou 215011, China; wangqinxin.wang@gmail.com; 3 Bioscience and Biomedical Engineering Thrust, Systems Hub, The Hong Kong University of Science and Technology (Guangzhou), Guangzhou 511453, China; ateng201@connect.hkust-gz.edu.cn; 4 Department of Computer Science and Engineering, Michigan State University, East Lansing, MI 48824, USA; 5 Department of Computational Medicine and Bioinformatics, University of Michigan, Ann Arbor, MI 48109, USA

**Keywords:** pairwise sequence alignment, multiple sequence alignment, protein monomer, protein complex, RNA, protein language model, function prediction, protein structure prediction, deep learning

## Abstract

Multiple sequence alignment (MSA) has evolved into a fundamental tool in the biological sciences, playing a pivotal role in predicting molecular structures and functions. With broad applications in protein and nucleic acid modeling, MSAs continue to underpin advancements across a range of disciplines. MSAs are not only foundational for traditional sequence comparison techniques but also increasingly important in the context of artificial intelligence (AI)-driven advancements. Recent breakthroughs in AI, particularly in protein and nucleic acid structure prediction, rely heavily on the accuracy and efficiency of MSAs to enhance remote homology detection and guide spatial restraints. This review traces the historical evolution of MSA, highlighting its significance in molecular structure and function prediction. We cover the methodologies used for protein monomers, protein complexes, and RNA, while also exploring emerging AI-based alternatives, such as protein language models, as complementary or replacement approaches to traditional MSAs in application tasks. By discussing the strengths, limitations, and applications of these methods, this review aims to provide researchers with valuable insights into MSA’s evolving role, equipping them to make informed decisions in structural prediction research.

## 1. Introduction

Multiple sequence alignment (MSA) is the process of aligning three or more biological sequences, typically protein, DNA, or RNA, to identify regions of similarity. These alignments are essential for inferring evolutionary relationships through phylogenetic analysis and highlighting homologous features between sequences. MSA also reveals mutation events, such as point mutations, insertions, and deletions, which help assess sequence conservation and infer the presence and function of protein domains, as well as secondary and tertiary structures.

Traditionally, MSA is used to compare biological sequences to identify similarities and differences, helping researchers study conserved regions, functional characteristics, and evolutionary relationships. However, in structural prediction—also known as homology-based sequence alignment—MSA plays a more specialized role. This approach allows users to input a target sequence, search large-scale protein databases, and identify homologous sequences for structure prediction.

MSA is fundamental to protein structure prediction. Sequence profiles—such as Position-Specific Scoring Matrices (PSSMs) and profile Hidden Markov Models (HMMs)—are typically derived from MSAs and are crucial for detecting homologous proteins and identifying conserved regions. In template-based modeling (TBM) for structure prediction, methods such as LOMETS [1] and HHpred [2] utilize these profiles generated from MSA for homology modeling (comparative modeling) and threading (fold recognition), enabling the identification of structural templates and facilitating the modeling of the target protein’s structure. Additionally, MSAs allow for the extraction of coevolutionary information to aid in structure prediction. For example, contact-based structure prediction methods, such as CONFOLD2 [3], utilize the MSAs generated from database searches of the query sequence to predict contact maps, which guide folding simulations to achieve accurate structure prediction. End-to-end methods, such as AlphaFold2 [4], also utilize MSAs as input data. These methods employ neural networks, specifically self-attention transformers and structural modules, to bypass complex folding simulations and directly achieve high-precision structure prediction.

Beyond structure prediction, MSA, as one of the most extensively utilized modeling techniques in biology, has broad applications across various fields, particularly in functional prediction. Sequence profiles, such as PSSMs and HMM profiles, generated from MSA contain rich evolutionary information. This makes them valuable for applications that have been widely explored and studied, such as Gene Ontology (GO) functional annotation, protein–ligand binding site prediction, protein post-translational modifications (PTMs) prediction, DNA/RNA binding site prediction, and disordered protein/region prediction. By leveraging only protein sequence alignments, InterProScan [5] enables the identification of potential functional domains, conserved regions, family members, and GO functional annotations. GO is a framework for representing how genes, in an evolving context, encode biological functions at the molecular, cellular, and tissue system levels. In the MetaGO [6] algorithm, sequence and sequence profile matching are employed for the identification of homologous sequences. In NsitePred [7], the PSSM profile generated by PSI-BLAST [8], along with structural features, is used as input for a support vector machine (SVM) classifier to predict protein-ligand binding sites. Similarly, S-SITE [9] combines PSSMs and Position-Specific Frequency Matrices (PSFMs) to represent template profiles for template recognition and complementary binding site prediction. In GlycoEP [10], PSSM is used as one of the features to predict N-, O-, and C-linked glycosylation sites using an SVM. It is noteworthy that PSSMs can also be used to predict binding sites in DNA- and RNA-binding proteins, as exemplified by tools such as DP-Bind [11] and RBPmap [12]. Moreover, features extracted from HMM profiles have been shown to further improve prediction accuracy in the field of DNA-binding protein prediction compared to PSSM features, as demonstrated by tools like HMMPred [13] and HMMBinder [14]. PSSM and HMM profiles can also be used as input features for machine learning classifiers to identify functional regions in intrinsically disordered proteins (IDPs), which lack stable 3D structures and exhibit dynamic interactions and diverse functions in biological processes. For example, molecular recognition features (MoRFs) are short sequences that undergo disorder-to-order transitions upon specific binding, with relevant analytical methods such as MoRFpred [15] and the approach proposed by Ronesh Sharma [16].

As sequencing technologies advance and the amount of available sequence data grows exponentially, the role of MSA will continue to be pivotal in decoding the complexities of biological systems. However, MSA construction has constraints, including a time-intensive process [17], labor-intensive manual design, and quality limitations for certain targets. With the advancement of deep learning technology, protein language models (PLMs) can not only be directly used to generate MSAs, but more importantly, they are now being employed to extract features from protein sequences as an alternative to MSAs in various application tasks.

In this work, we provide an overview of the history of constructing MSA for protein monomers, protein complexes, and RNA. For protein monomers, methods include sequence-based approaches represented by FASTA [18], HMM-based approaches represented by SAM [19], *k*-mer-based approaches represented by MMseqs2 [20], multi-stage hybrid approaches represented by DeepMSA2 [21], and deep learning-based approaches represented by pLM-BLAST [22]. For protein complexes, methods include genomic distance-based approaches represented by EVcomplex [23], phylogeny-based approaches represented by ComplexContact [24], protein–protein interaction-based approaches represented by cpxDeepMSA [25], PLM-based approaches represented by ESMpair [26], and hybrid approaches represented by DeepMSA2-Multimer [21]. MSA construction methods for RNA include sequence-based approaches such as BLASTn [27], HMM-based approaches such as nhmmer [28], covariance model (CM)-based approaches such as Infernal [29], and hybrid approaches combining multiple approaches like RNAlien [30]. Finally, we discuss alternative methods to MSA in application tasks, namely PLM-based methods, which include methods that use MSA as input like MSA transformer [31], autoencoding methods with single-sequence input like ESM-1b [32], autoregressive methods with single-sequence input like ProtGPT2 [33], and methods based on alternative frameworks like ProtT5 [34].

Table 1 provides links to and classifications of the methods discussed in this work, while Table 2 summarizes the advantages and limitations of each type of method for ease of reference.

## 2. An Overview of Multiple Sequence Alignment

### 2.1. Multiple Sequence Alignment for Protein Monomer

Many proteins can function in their monomeric form. Therefore, constructing an MSA of monomeric proteins is crucial for understanding their structure and function, and provides a foundation for subsequent in-depth studies of protein complexes. Pairwise alignment based on dynamic programming serves as the foundation for subsequent algorithms, which can be further improved to enable homology sequence search. Additionally, there are methods specifically designed for fast and sensitive homology sequence detection. Multiple sequence alignment for protein monomers primarily includes sequence-based methods, HMM-based methods, *k*-mer-based techniques, hybrid approaches, and deep learning-based strategies.

#### 2.1.1. Dynamic Programming-Based Pairwise Alignment

The Needleman–Wunsch algorithm (NWalign) from 1970 and the Smith–Waterman (SWalign) algorithm from 1981 represent early classic applications of dynamic programming to the comparison of biological sequences, serving as foundational concepts for subsequent alignment algorithms.

NWalign [35] is a heuristic algorithm for detecting homologous sequences through global sequence alignment, which first introduced the iterative matrix calculation method to optimize alignment results based on the dynamic programming algorithm. In particular, a penalty scoring scheme is initially defined, encompassing scores for matching or mismatching positions between two sequences, along with penalties for gaps. This serves as the foundation for constructing a scoring matrix. Subsequently, through the process of backtracking the scoring matrix, the globally optimal path, representing the optimal matching sequence, is determined. NWalign prioritizes the comprehensive examination of sequence similarity and alignment across the entire length. However, this emphasis may pose challenges in detecting distantly conserved homologous relationships that depend on short subdomain fragments [36].

SWalign [37] is optimized on the basis of the NWalign, which enhances its applicability to local homologous sequence alignment. The primary improvement of the SWalign lies in truncating the values of the score matrix to zero, thus preventing the occurrence of negative numbers within the matrix. During traceback, the algorithm initiates from the highest-scoring matrix element and terminates upon encountering a cell with a value of zero, resulting in the generation of highly similar local alignment sequences.

Incorporating PLM embeddings (the details are provided in Section 2.4) into dynamic programming-based pairwise alignment has been shown to improve alignment performance. For example, PEbA [38] generates substitution matrices from ProtT5 embeddings using scaled cosine similarity. The alignment parameters applied were −11 for gap opening and −1 for gap extension in local alignments. This approach significantly outperforms pairwise alignments based on conventional scoring matrices, yielding varying degrees of improvement in alignment quality for sequence pairs with differing levels of similarity. EBA [39] computes the Euclidean distance of amino acid pair embeddings as a scoring matrix and employs an efficient signal enhancement procedure, facilitating a more effective comparison of representations. The quality of its alignments is comparable to that of the structural alignment method Foldseek [40].

Alignments based on dynamic programming ensure the optimal outcome for pairwise sequence alignments. However, this approach also leads to a high time complexity of *O*(*m* · *n*), where *m* and *n* denote the lengths of the two sequences being aligned. Consequently, when the sequences are particularly long, the computational time increases significantly, along with substantial memory consumption.

#### 2.1.2. Multiple Sequence Alignment

When applying dynamic programming algorithms directly to the multiple sequence alignment, the time complexity grows exponentially with the increase in the number of sequences, specifically *O*(*2^m^* · *n^m^*), where *m* represents the number of sequences and *n* denotes the sequence length. The immense computational burden renders the straightforward use of standard dynamic programming methods impractical in real-world applications. Even with the reduction in time complexity to *O*(*n^m^*) [41] based on the sum of all pairs (SP) score scheme [42], the problem has still been proven to be NP-complete [43].

Therefore, heuristic algorithms are commonly used to tackle large-scale and diverse MSA problems, allowing for quick approximate solutions. The progressive alignment algorithm is the most popular, simple, and effective heuristic method, classically proposed by Feng and Doolittle [44], consisting of three steps: (1) using pairwise alignment methods to compare all sequences and obtain similarity scores; (2) generating a guide tree from the similarity (or distance) matrix; and (3) starting with the two most similar sequences and progressively adding new sequences according to the guide tree until all sequences are included. This approach has the advantages of shorter computation time and lower memory usage. Classic progressive methods like ClustalW [45] utilize scoring functions based on general amino acid substitution models, demonstrating rapid performance and yielding reasonable results for relatively similar sequences (e.g., with sequence identity above 30%). To correct or reduce errors introduced during the progressive alignment steps, MAFFT [46] and MUSCLE [41] rely on iterative optimization to enhance alignment quality. This strategy is applied after the initial progressive assembly of multiple sequences, where aligned sequences are repeatedly divided into sub-alignments and realigned. T-Coffee [47], on the other hand, was the first to propose reducing errors by exploring consistency information in progressive alignments; specifically, the scoring function for two sequences takes into account not only their pairwise alignment results but also the alignment information from other sequences, which is incorporated into the consistency measures. However, progressive alignment algorithms perform poorly under conditions of low sequence consistency. The vector-clustering Multiple Sequence Alignment (vcMSA) [48] method clusters amino acid embeddings generated by PLMs and subsequently employs graph-theoretic approaches to establish a consistent ordering of MSA columns. The high-dimensional contextual embeddings encapsulate higher-order structural and functional information, and the incorporation of this additional data enhances the accuracy of the alignments.

The co-evolutionary information embedded in MSA can be utilized for phylogenetic tree reconstruction, making it an important downstream task of MSA. For instance, the PHYML [49] program estimates large phylogenies using maximum likelihood (ML), which is fundamentally based on a simple hill-climbing algorithm. Similarly, IQ-TREE [50] performs phylogenetic inference through ML, employing a more efficient approach that combines elements of hill-climbing algorithms, random perturbations of the current best trees, and extensive sampling of initial starting trees. The Molecular Evolutionary Genetics Analysis (MEGA) [51,52,53,54] software includes numerous sophisticated methods and tools for phylogenomics and phylomedicine, supporting five distinct methods for constructing evolutionary trees: ML, Neighbor-Joining, Minimum Evolution, unweighted pair-group method with arithmetic means (UPGMA), and Maximum Parsimony. Umberto Lupo et al. employed a PLM, MSA Transformer, trained on MSA, where the column attention heads effectively capture Hamming distances, thereby encoding phylogenetic information [55].

The aforementioned methods represent traditional pairwise alignment and conventional MSA approaches and applications. The subsequent heuristic MSA algorithms all support database searches for homologous sequences, some of which focus on improving alignment methods in the context of database search, while others are specifically designed for homolog detection, emphasizing updates in search algorithms.

#### 2.1.3. Sequence-Based Approaches for Protein Monomer’s MSA

**Figure 1:**
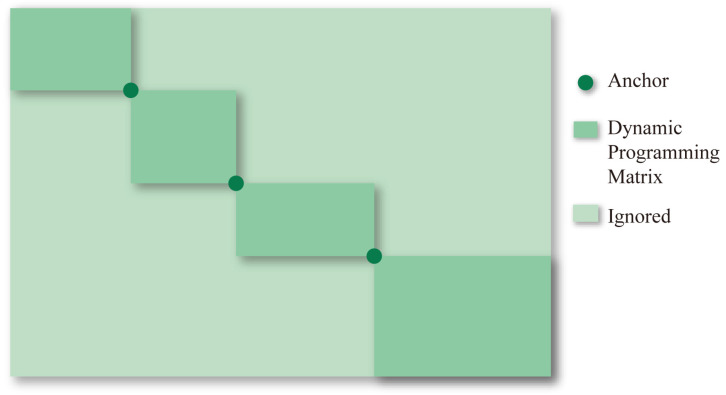
Homologous segments (or seeds) are used as ‘anchors’, and each anchor point divides the dynamic programming matrix into four submatrices located at the four corners. The submatrices positioned at the lower left and upper right are ignored.

The sequence-based algorithms introduced in this section are all improvements upon pairwise alignment methods, enabling database search functionality. To solve sequence alignment of very long sequences, heuristic algorithms are considered. The most widely used method is to limit state transitions and conduct the alignment within a smaller search space. FASTA and Basic Local Alignment Search Tool (BLAST) are two heuristic classic alignment algorithms based on divide-and-conquer. These methods are designed to find seeds (homologous segments) to search protein and DNA databases for sequence similarities. Seeds act as anchor points to divide the dynamic programming matrix into four submatrices located at the four corners. The dynamic programming matrix will be reduced if more anchor points distributed throughout the sequences are discovered, allowing for a reduction in time and space complexity, as shown in Figure 1 [56].

In 1985, Pearson et al. first designed the FASTP [57] program for searching protein sequence libraries to build alignments, the algorithm encompasses three fundamental stages. Firstly, it employs a lookup table [58] to find all identities or groups of identities between two protein sequences and the output is the 10 best diagonal regions found by a simple formula based on the number of ktup (a parameter for determining how many consecutive identities are required in a match) matches and the distance between the matches without considering shorter runs of identities, conservative replacements, insertions or deletions [57,59]. Secondly, by rescoring the 10 best regions using the PAM250 matrix, which allows for conservative replacements and enables runs of identities shorter than ktup to contribute to the similarity score, the output of this step consists of the best initial regions evaluated by the PAM250 matrix. Finally, FASTP uses a modification of the optimization method described by NWalign [35] and SWalign [37] to align the sequences with the highest scores. Subsequently, the FASTA [18] program, introduced in 1988, implemented two key advancements upon the foundation of FASTP. The first enhancement allows the use of a scoring matrix based on the genetic code for DNA sequence alignment, facilitating easy customization of alignment tasks by adjusting the similarity scoring matrix and gap penalties. The second improvement involves checking whether several initial regions are joined together and calculating the optimal alignment of initial regions that can be joined to form a single alignment, with locations of initial regions, respective scores, and a gap penalty. This enables the FASTA program to increase sensitivity without a large loss of selectivity or decrease in speed. Even though the FASTA program utilizes rigorous algorithms at each step with a realistic model of evolution, it is heuristic due to its hierarchical nature. Moreover, apart from the PAM matrix, a variety of different substitution matrices have been developed over the years. For instance, the widely used Blocks Substitution Matrix (BLOSUM) family of matrices [60]; the variable time maximum likelihood (VTML) substitution matrices, proposed by Muller et al., which are based on divergent alignments for identifying distantly related protein sequences [61]; and a matrix introduced by Yamada and Tomii, which utilizes principal component analysis and the variabilities across existing substitution matrices [62]. Additionally, some methods derive substitution matrices from structural information. For example, Prlic et al. developed a substitution matrix based on a set of protein structures with high structural similarity but low sequence identity [63]. Another approach, the ProtSub matrix [64], incorporates structural information and filters out irrelevant residue pairs by retaining only those that are spatially close, significantly improving protein sequence alignments by reducing false positives.

Similar to the FASTA program, early versions of BLAST [65] confine dynamic programming to a banded section of the full path graph, encompassing regions of identified similarity, thus facilitating a trade-off between speed and sensitivity. BLAST initially partitions the input sequence into discrete “seed words” of length *w* (typically 3 for proteins and 11 for nucleotides). It then swiftly identifies pertinent candidate sequences and their specific positions within these sequences through the utilization of a pre-established indexing table. This process is iteratively executed for all seed words, resulting in a hit map that delineates the correspondence between the query sequence and the candidate sequences. Subsequent bidirectional extensions are conducted until the aggregate score falls below a predetermined threshold. Ultimately, a classical dynamic programming approach is employed within the extended regions to ascertain the definitive alignment outcome. In 1997, Lipman et al. reported Gapped BLAST and PSI-BLAST as a new generation of protein database search programs [8]. The new version of BLAST has been optimized in three aspects, significantly enhancing computational speed and achieving higher sensitivity. Firstly, it increased the search speed with the two-hit method. In contrast to the old BLAST, the two-hit method only requires two non-overlapping word pairs on the same diagonal for extension. Therefore, with a smaller number of hits for extension, the average amount of computation decreases, and the speed increases. The threshold parameter *T* (a higher value of *T* leads to higher speed and an increased probability of missing weak similarities) is lowered in the new version for more hits, which increases the sensitivity and speed. Secondly, the new version of the program gained the ability to construct gapped alignments, using dynamic programming to extend a central pair of aligned residues in both directions. Different from the original BLAST, the new version of Gapped BLAST considers only alignments that drop in a score of no more than *Xg* below the best score yet. Therefore, this approach adapts the region of the path graph explored to the data, making the search more efficient and sensitive. Finally, the new version of PSI-BLAST is reported as a new method for multiple alignment construction. PSI-BLAST takes a PSSM generated by a BLAST search from significant alignments in round *i* as an input for round *i*+1. PSI-BLAST utilizes motif or profile search methods for a more sensitive result of distant relationships between sequences.

In conclusion, BLAST and FASTA are two tools for pairwise sequence alignment in bioinformatics, used to search for similarities between DNA or protein sequences. BLAST is widely employed for local alignment of nucleotide and amino acid sequences. FASTA serves as a refined tool for similarity searching, utilizing sequence patterns or words, particularly suited for comparing less similar sequences. The primary distinction between BLAST and FASTA lies in their respective strategies for similarity searching.

To address computational bottlenecks in metagenomics and data-intensive evolutionary projects, DIAMOND [66] has been proposed as a highly suitable tool for aligning translated DNA sequences with protein sequence reference databases in high-throughput environments. DIAMOND employs the traditional sequence alignment ’seed-and-extend‘ paradigm, incorporating additional techniques such as reduced alphabet usage, spaced seeds, and double indexing to achieve efficient search and alignment within large-scale databases. Compared to previous algorithms, DIAMOND integrates these advanced methods to enhance performance and sensitivity in extensive sequence comparisons. Specifically, seed matches will be extended to full alignments between the queries and references, shorter seeds contribute to sensitivity, while longer seeds enhance speed. To increase speed without losing sensitivity, DIAMOND has employed a new alphabet reduced to a size of 11 letters. Another approach to improving seed steps is employing spaced seeds, where longer seeds are used while only specific positions are considered. The quantity and precise arrangement of these positions are referred to as the weight and shape of the spaced seed, respectively. By appropriately selecting the shape of spaced seeds [67], sensitivity can be improved. One drawback of utilizing multiple spaced seeds is the significant memory consumption. To address this issue, DIAMOND adopts a solution where it constructs and processes indexes for one seed shape at a time, then releases the memory used by the previous seed shape before transitioning to the next one. Unlike most seed-and-extend programs, which typically build an index structure only on the reference sequences (such as a hash table or Ferragina–Manzini Index), DIAMOND employs a double-indexing approach, involving indexing both the queries and the reference sequences. In DIAMOND, an index comprises a sorted list of seed-location pairs based on a compressed representation of the seed. By simultaneously traversing these two indices lists linearly, the algorithm can identify all matching seeds between the query and reference sequences. This process enables local alignment computations at the corresponding seed locations. What is more, the double-indexed approach also leads to a linear approach memory access pattern.

#### 2.1.4. HMM-Based Approaches for Protein Monomer’s MSA

**Figure 2:**
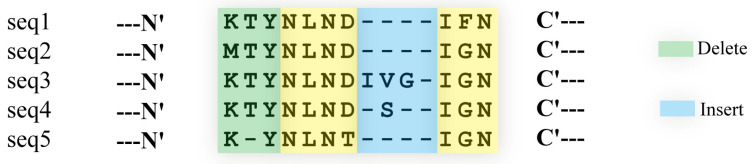
Profile HMM for the following sequences. In MSA, conserved regions are identified based on a threshold of more than 75% similarity, shown in green and yellow. In Figure 2, there is a gap in a conserved region, which is indicated by a delete state. Delete states in a profile HMM account for deletions (nucleotides or amino acids) in conserved regions, while insert states account for insertions in non-conserved regions. This distinction is crucial for accurately modeling sequence variability and conservation.

Tools like BLAST [65], which performs pairwise sequence alignment, assess sequence similarity by calculating the optimal alignment score. However, when detecting distant homology in protein families, MSA methods based on HMMs, such as SAM, HMMER, HHblits, and HHsearch, have proven to be more effective [68,69], as illustrated in Figure 2. These models differ from pairwise alignment in that they employ probabilistic states to determine the frequencies of specific residues (amino acids or nucleotides) at particular positions within MSAs, and model the transition probabilities between states representing matches, insertions, and deletions. Moreover, profile HMMs incorporate a scoring mechanism to compare query sequences against the model, assigning scores that facilitate homology recognition and potential for structure prediction [70].

SAM [19] is a comprehensive software suite specifically designed to analyze biological sequences using profile HMMs, its core functions focus on constructing, optimizing, and applying profile HMMs for sequence analysis and homology detection in proteins and nucleic acids. SAM utilizes a linear HMM where each state corresponds to a column in an MSA. This thoughtful design effectively considers potential insertions and deletions at each position during sequence–profile HMM alignments, enabling SAM to adeptly capture distant homologies. After constructing the profile HMM models, SAM employs the Viterbi and Forward algorithms to compute the similarity between sequences and the profile HMMs. The Viterbi algorithm is used for decoding in model inference and is based on dynamic programming to find the shortest path for a sequence. Specifically, the algorithm identifies the most probable path through the profile HMM for an observed sequence, calculating the log-odds score at each step. This score represents the likelihood that the observed sequence matches a null model, with higher scores indicating stronger alignments. By maximizing this log-odds score, the algorithm enhances alignment accuracy, ensuring the best possible match. Meanwhile, the Forward algorithm computes the probability of an observation sequence in an HMM through a recursive process. Specifically, the algorithm sums probabilities across all paths, offering a comprehensive likelihood of alignment and significantly boosting the ability to detect distant homologies. This process is used to search databases for sequences and to assess their similarity to the models. Notably, SAM includes a script ‘target99’ [71], analogous to the principle of PSI-BLAST [8], which enables iterative searching of sequence databases to automatically generate MSAs.

Similar to SAM, HMMER is a rapid heuristic algorithm that also employs profile HMMs for sequence alignment and the detection of sequence homology, primarily in protein and nucleic acid analyses. Essentially, its core principle involves constructing profile HMMs to capture patterns in sequences, utilizing these models to search databases for sequences with high similarity. Additionally, HMMER shares the same computational strategies as SAM, the Viterbi and Forward algorithms. Moreover, the inclusion of ‘sparse rescaling’ in HMMER3 [72] prevents numerical underflow. These techniques complement each other and collectively elevate HMMER’s sensitivity and effectiveness in identifying sequence homology, particularly with distant homology.

Although both SAM and HMMER are robust tools based on profile HMMs, there are distinct differences between them, as presented in Madera’s research [73]. In core function, SAM has the capability to automatically generate MSAs, whereas HMMER includes model-scoring programs that SAM does not. In terms of processing speed, HMMER is faster than SAM when dealing with large databases, but SAM performs better with small databases. In model evaluation, SAM excels with high-quality and diverse alignments, while HMMER is more effective with lower-quality alignments. Additionally, there are differences in user-friendliness and other aspects. Overall, users can choose between SAM and HMMER based on their specific needs.

**Figure 3:**
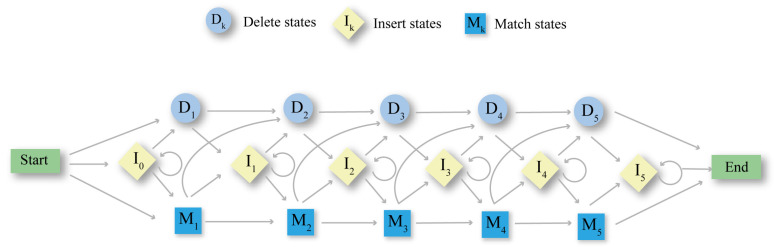
Principles of Profile HMM. The model consists of three types of states: Match (M), Insert (I), and Delete (D). Arrows indicate possible transitions between states, capturing the variability and conservation patterns across sequences. This model facilitates the accurate representation of sequence alignments and the identification of evolutionary conserved elements.

The log-odds score has been widely established for identifying homology recognition in sequence-HMM and sequence-profile comparisons like HMMER and SAM [74]. Building on this foundation, HHsearch extends the concept of the log-odds score to HMM-HMM comparisons by introducing the log-sum-of-odds score, which quantifies the probability of co-emission of aligned paths from two profile HMMs. This process involves dynamic programming to compute the maximum log-sum-of-odds score via the Viterbi algorithm in HMM-HMM aligned paths, thereby enabling the detection of sequence homologies across a broad range of evolutionary distances, as shown in Figure 3. Moreover, HHsearch improves alignment quality by integrating predicted secondary structure information, thereby setting new standards for the sensitivity and accuracy of alignment tools.

**Figure 4:**
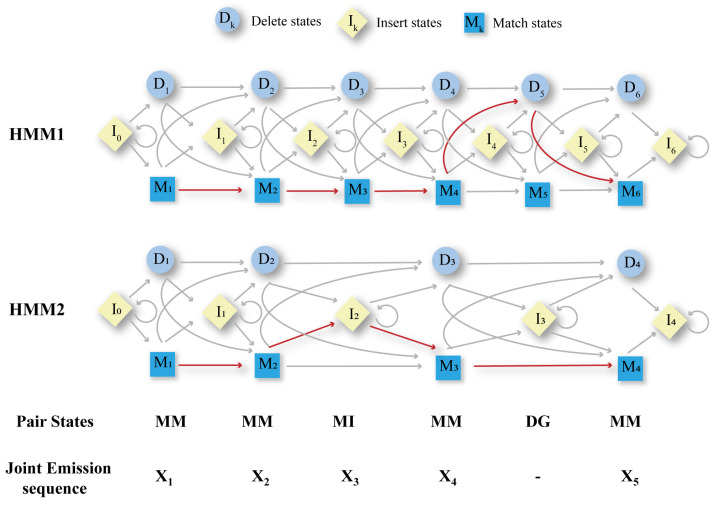
Profile HMM aligns to profile HMM. It contains the comparison between two profile HMMs, which is the core idea of HHblits, HHsearch, and other programs. In contrast to the transitions between states (M, I, D) within a single profile HMM, HMM–HMM introduces pair states that describe the combined states and their relationships within the two models during the alignment process. Notably, the “DG” state represents a Delete–Gap pairing at a specific alignment position. One profile HMM is in a Delete (D) state, meaning it skips this position without emitting any symbol, while the other profile HMM is in a Gap (G) state, indicating a gap at this position. This provides more complex and detailed sequence alignment information. The Joint Emission sequence represents the paired sequences emitted simultaneously by the two HMM models during alignment.

HHblits [75] is an iterative sequence search tool using profile HMM-profile HMM comparisons, a core technique pioneered by HHsearch [76], to perform fast and sensitive searches of sequence databases like NCBI’s nonredundant (nr) database or Universal Protein Resource (UniProt). While maintaining the same high sensitivity as HHsearch, HHblits performs faster searches than traditional tools like PSI-BLAST [8]. This enhanced performance is not only due to its iterative HMM–HMM search methodology, which builds upon the foundation laid by HHsearch but also the integration of context-specific pseudo-counts and a fast prefiltering mechanism using discrete states [75]. The context-specific pseudo-counts enhance the accuracy of model predictions under various sequence conditions, while the discrete state prefilter significantly accelerates the search process by simplifying the initial screening of potential matches, as shown in Figure 4. These innovations enable HHblits to efficiently and rapidly search through extensive databases. Overall, the emergence of HHblits represents a significant advancement in tools for constructing protein MSA. With its innovative features, this tool can swiftly and accurately identify homologies within extensive protein databases. Consequently, it is widely employed in areas such as structure prediction and functional annotation of proteins, facilitating deeper insights into protein functions and evolutionary relationships.

#### 2.1.5. *k*-Mer-Based Approaches

**Figure 5:**
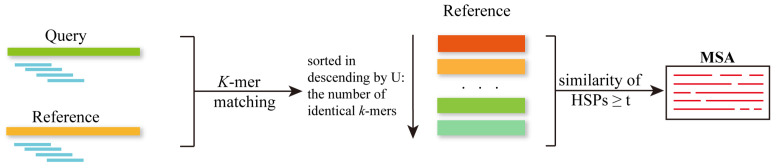
USEARCH utilizes a heuristic approach to quickly find the most likely matches. It builds an index based on the *k*-mer of the reference sequence, matches the *k*-mer between the query and the reference sequence, relies on a unique word count (U-value) to sort and filter the sequences, and refines the matching based on *k*-mer similarity.

USEARCH [77] is a unique sequence analysis algorithm based on pairwise alignment for sequence database searching. Its uniqueness lies in employing a heuristic approach to rapidly identify one or a few promising hits, rather than exhaustively searching for all homologous sequences, as shown in Figure 5. This approach helps reduce the resources required. Similar sequences often share similar short words, known as *k*-mer, with a fixed length of *k*. USEARCH generates a metric called U, representing the number of unique words shared between the query and the database sequences. Clearly, this vector is positively correlated with the similarity between sequences [78]. Hence, in this algorithm, target sequences are sorted in descending order based on their unique word count U. If a target sequence exists with similarity to the query satisfying the threshold, it is more likely to be found at the beginning of this sorted list. Therefore, the target sequences are compared to the query in descending order of U. If a target sequence meets or exceeds the predetermined similarity threshold, it is accepted; otherwise, it is considered a failed match. (i) If an acceptance happens, it is likely to be found among the initial few targets tested. (ii) The first acceptance is likely to have the highest possible similarity or be close to it. (iii) As the number of failed attempts increases, the probability of finding high-similarity matches in the database decreases rapidly. The search ends with a predetermined number of acceptances or rejections. Explicit sequence comparisons begin with finding gapless high-scoring segment pairs (HSPs). For USEARCH, HSPs are identified as spaced pairs of matching words of length *k*. If the similarity of the HSPs is <*t* then the target is rejected. Otherwise, after using banded dynamic programming [79] to align the remaining regions, similarity can be computed from the final alignment. As for E-values, the Karlin-Altschul statistics [79] are employed.

**Figure 6:**
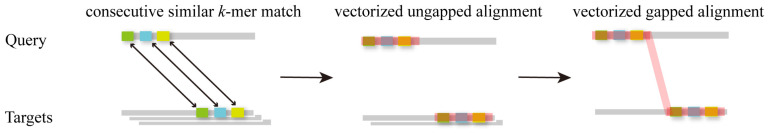
MMseqs2 consists of three stages: a short word (‘*k*-mer’) match stage, vectorized ungapped alignment, and gapped alignment. Sensitivity gradually increases to ensure efficient and accurate sequence matching.

To achieve sensitive searches of sequences within massive data sets, Martin Steinegger and Johannes Söding have developed MMseqs2 [20], a parallelized and open-source software suite tailored for the precise searching based on pairwise alignment and clustering of extensive protein and nucleotide sequence repositories. In MMseqs2 searching, three stages are involved in finding similar sequences in the target database, progressively increasing in sensitivity: a short word (‘*k*-mer’) match stage, vectorized ungapped alignment, and gapped alignment, as shown in Figure 6. The key improvement in the prefiltering stage lies in combining the double-match criterion with maximizing the length of *k*-mers. On the one hand, MMseqs2 identifies matches between similar *k*-mers rather than solely detecting exact *k*-mer matches, unlike most fast tools such as DIAMOND [66] and USEARCH [77]. On the other hand, the final decision is based on 2 × 7 = 14 residues, as opposed to just 2 × 3 in BLAST or the 11-letter size of DIAMOND’s alphabet. This enables MMseqs2 to maintain efficiency while considering more sequence information. MMseqs2 achieves accelerated searching through parallelization on three levels: critical time-sensitive components are manually vectorized, queries can be distributed across multiple cores, and the target database can be partitioned into chunks distributed to multiple servers. What is more, MMseqs2 effectively suppresses false-positive matches between locally biased segments to compensate for some unavoidable loss of sensitivity due to its heuristic prefilters.

#### 2.1.6. Multi-Stage Hybrid Approaches to Search Metagenome

The sequence-based, HMM-based, and *k*-mer-based MSA methods discussed earlier were not specifically designed for three-dimensional structure prediction. While applicable to structure predictions to some extent, these methods face the challenge of excessive search time when handling large datasets. The methods introduced in this section, however, are specifically developed to enhance the prediction of long-range homologous contacts and folding recognition. They are designed for database search rather than improvements based on pairwise alignment algorithms. The MSAs constructed by these approaches significantly improve the accuracy of protein tertiary structure prediction.

Traditionally, the construction of high-quality MSAs has largely relied on genomic databases from individual species such as humans, mice, or yeast. David et al. [80] pioneered the integration of diverse metagenomic sequence data into sequence alignments by using the ‘HMMsearch’ tool from the HMMER package with each Pfam HMM as the query against the Integrated Microbial Genomes (IMG) database. This approach significantly enhanced the accuracy of subsequent structural predictions. This indicates that incorporating metagenome sequence data into the construction of MSAs can significantly enhance the diversity of protein sequences, enrich the heritable variation, and provide a solid foundation for coevolutionary analysis.

**Figure 7:**
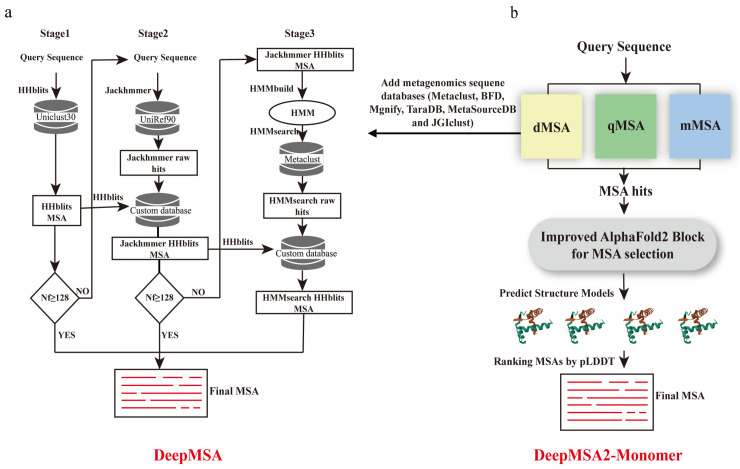
The pipeline of DeepMSA and DeepMSA2-Monomer. (**a**) The DeepMSA algorithm is divided into three stages. In the first stage, HHblits is used to search the UniClust30 database. If the sequence count is insufficient and the normalized effective sequence count (Nf) is below 128, the second stage is initiated. In the second stage, JackHMMER searches the UniRef90 database, and full-length sequences are extracted using ‘esl-sfetch’ to construct a custom database, after which HHblits updates the multiple sequence alignment (MSA). If the Nf in the second stage is higher, it replaces the MSA from the first stage. If Nf remains below 128, the third stage is performed, where the MSA is converted into a hidden Markov model (HMM) and searched in the Metaclust database using HMMsearch, followed by final MSA generation using HHblits. (**b**) DeepMSA2-Monomer incorporates large genomic and metagenomic sequence databases and, building upon DeepMSA, integrates dMSA, qMSA, and mMSA to generate multiple MSAs. It then employs a deep learning-driven MSA scoring strategy, simplified from AlphaFold2, for optimal MSA selection.

In 2019, a new MSA construction method was introduced as DeepMSA [81]. In contrast to conventional methods that utilize a singular approach for MSA construction, DeepMSA integrates multiple specialized tools to facilitate rapid and highly sensitive exploration of metagenomic databases. The algorithm is structured into three distinct phases (Figure 7a). In Stage 1, HHblits is used to search the UniClust30 database. If Stage 1 generates insufficient sequences, with the normalized number of effective sequences (Nf) being less than 128, Stage 2 is initiated. In Stage 2, JackHMMER [82] searches the UniRef90 database. Afterward, ‘esl-sfetch’ from the HMMER package is used to extract full-length sequences from the previous hits to build a custom HHblits format database. HHblits is then applied to search this custom database, starting from the MSA generated in Stage 1. If the MSA from Stage 2 has more Nf than Stage 1, it replaces Stage 1’s MSA. If previous stages yield low sequence numbers, that is, if Nf is less than 128, Stage 3 is executed. The MSA from the preceding stage is converted into an HMM using ’HMMbuild’ from the HMMER package. This HMM is searched against the Metaclust metagenome sequence database using HMMsearch. Hits from HMMsearch are used to construct a new custom HHblits database, which is then searched by HHblits to generate the final MSA.

For further improvement, Zheng et al. reported the DeepMSA2 [21] pipeline in 2023, which demonstrated excellent performance in Critical Assessment of protein Structure Prediction 15 (CASP15) experiments. Compared to the former DeepMSA, DeepMSA2 is based on a huge genomics and metagenomics sequence databases containing a total of 40 billion sequences. DeepMSA2-Monomer is specially designed for protein monomer MSA construction. In detail, DeepMSA2-Monomer utilizes six metagenomics sequence databases, including three third-party databases (Metaclust, BFD, and Mgnify) and three in-house databases (TaraDB, MetaSourceDB, and JGIclust). Moreover, DeepMSA2-Monomer couples several new MSA generation pipelines, including dMSA, qMSA, and mMSA, to create multiple MSAs (Figure 7b). Then, a deep learning-driven MSA scoring strategy simplified from AlphaFold2, is employed for ranking MSA. In this simplified version, the template detection module is deactivated, and the embedding parameter is set to one, allowing for rapid model generation. At most, 10 MSAs will be given and will be taken as input of a modified AlphaFold2 program for five structure modeling. The highest Predicted Local Distance Difference Test (pLDDT) score among the five structures will be the rank score of the MSA. The pLDDT measures the confidence in the local structure, reflecting the consistency between each amino acid residue in the predicted structure and the experimental structure. The final MSA is the one with the highest rank score among all created MSAs. In contrast to previous MSA construction programs, such as HHblits, PSI-BLAST, and JackHMMER, the DeepMSA2 package improves the accuracy of contact and secondary structure predictions. Meanwhile, the integration of huge metagenomic datasets combined with the application of a new deep-learning-driven MSA scoring strategy increases the accuracy of MSA construction and also hints at the solution of protein tertiary structure predictions.

The MSA construction component of the Yang–Server [83] structure prediction method, proposed by Yang et al., also employs a multi-stage hybrid approach. It leverages complementary sequence databases and three advanced search algorithms to generate high-quality MSAs. Firstly, HHblits is used to search against three HMM profile databases, including UniClust30, UniRef30, and BFD. The top MSA is determined by the average probability of the top 15L residue pairs in the predicted distance map [84]. Secondly, they use MMseqs2 to search against UniRef30 and colabfold_envdb [20]. The first two methods are sufficient for easy targets. The third method is designed for challenging targets. They use jackhammer [85] to search against the FASTA database for sequence relatives. Full-length hits are selected for forming a database of candidate homologues, which is later converted into an HMM profile database by UniClust [86]. Then, HHblits is utilized to search against this HMM profile database to generate MSAs.

#### 2.1.7. Deep Learning-Based Approaches

**Figure 8:**
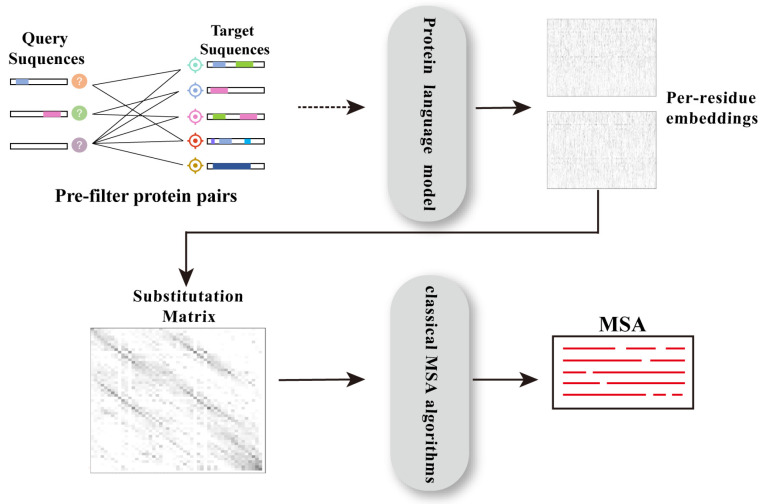
Schematic diagram of PLMs for MSA construction. Optional pre-filtering methods are used for selection, followed by representation of query-target pairs at the amino acid level using PLMs and calculation of substitution matrices. Finally, classic multiple alignment methods are employed.

The significant advancements in the field of Natural Language Processing (NLP) have provided potential solutions to many challenges encountered in protein research. By applying NLP techniques to protein sequences—treating them similarly to linguistic data—researchers have developed large-scale PLMs (the details of PLMs can be found in Section 2.4). These PLMs have achieved remarkable success in extracting biological information from protein sequences and have thus emerged as potential tools for constructing MSAs, either by directly generating MSAs using deep learning-based methods or by employing PLM-based embeddings as alternatives to sequence profiles in MSA searches (Figure 8).

The MSA-augmenter [87] represents a Transformer-based [88] seq2seq model tailored for homogeneous protein sequence generation. It excels in producing high-quality sequences essential for protein folding tasks, particularly when dealing with low-quality MSAs where homologous sequences of target proteins are scarce. To concurrently consider the global structural information within the input MSA, the MSA-augmenter leverages a tied-row and column attention mechanism, inspired by the MSA Transformer [31]. Moreover, supplementary cross-column and cross-row modules are integrated into the decoder, enabling the simultaneous generation of multiple sequences. This functionality facilitates the production of diversified and new co-evolutionary MSA results, thereby fortifying MSA and enhancing downstream protein structure prediction. Nevertheless, it is evident that this approach is currently constrained by the transformer’s limitations regarding sequence length, as well as the scale of the pre-trained model and database. Should advancements be made in these areas in the future, there exists considerable potential for enhancing the reliability of the results.

pLM-BLAST [22] integrates the representations from PLMs with the BLAST or PSI-BLAST [8] algorithm to detect homology between protein sequences, particularly for distant homology relationships. This method focuses more on optimizing pairwise alignment methods based on BLAST, rather than on improving the search algorithm itself. pLM-BLAST does not require training in a specialized deep learning model and can be combined with representations from any PLM. It generates a substitution matrix using the embeddings of two sequences to represent the cosine similarity between each pair of residues in the sequences. Unlike SWalign [37], pLM–BLAST does not apply gap penalties or truncate values to zero when creating the score matrix. This results in more severe penalties for dissimilar regions, thereby reducing the total number of potential alignments. Aside from local alignments, pLM–BLAST has the capability to conduct global alignments utilizing NWalign. The alignment accuracy can be on par with HHsearch [76], while significantly enhancing processing speed. However, in local mode, it tends to produce alignments that are shorter yet of higher precision compared to those generated by HHsearch, the evolutionary significance of which is yet to be explored.

PLMsearch [89] is a homologous protein search approach that leverages protein representations generated by PLMs as input. The search process of this method offers unique advantages, while the alignment process is based on an improved pairwise alignment approach. Differing from pLM–BLAST [22], this algorithm integrates a structural similarity prediction module for pre-filtering, thus avoiding numerous irrelevant low-similarity alignments. Moreover, it notably enhances sensitivity in identifying homologous query-target pairs characterized by low sequence consistency but high structural similarity. Initially, proteins in the target dataset sharing the same Pfam clan domain as the query protein are searched and paired with the query. Subsequently, utilizing a trained SS-predictor model, structural similarity is predicted using the PLM representation of each protein pair as input. Pairs with higher structural similarity are selected based on this criterion, and for those with significant similarity, PLMalign is employed for either global or local alignment of query-target pairs. In this regard, PLMalign utilizes dot product to replace the cosine substitution matrix and employs a linear gap penalty instead of an affine gap penalty, resulting in a faster alignment speed compared to pLM-BLAST. PLMsearch rivals MMseqs2 [20] in speed and matches state-of-the-art structural search methods in sensitivity, presenting a promising avenue for a more convenient large-scale homologous protein search approach.

MSA–augmenter, pLM–BLAST, and PLMsearch integrate PLMs into classical MSA algorithms, achieving innovative improvements in alignment and search strategies. With further advancements in PLM technology, they undoubtedly offer new perspectives for protein research that involves complex biological information, showcasing their unique advantages and significance.

### 2.2. Multiple Sequence Alignment for Protein Complex

Many proteins function in biological systems through interactions between different monomers or subunits, often forming complexes [90]. The prediction of the structure and function of individual monomers has reached a relatively high level of accuracy, there is now a growing focus on addressing the more intricate challenge of predicting the structure, interactions, and functional dynamics of protein complexes, where constructing MSAs for complexes remains a critical step. The prevailing strategy entails pairing individual MSAs that satisfy specific criteria to construct MSAs for protein complexes. This encompasses methodologies based on gene distance, phylogenetic inference, protein–protein interaction databases, PLMs, as well as integrative hybrid approaches.

#### 2.2.1. Genomic Distance-Based Approaches

**Figure 9:**
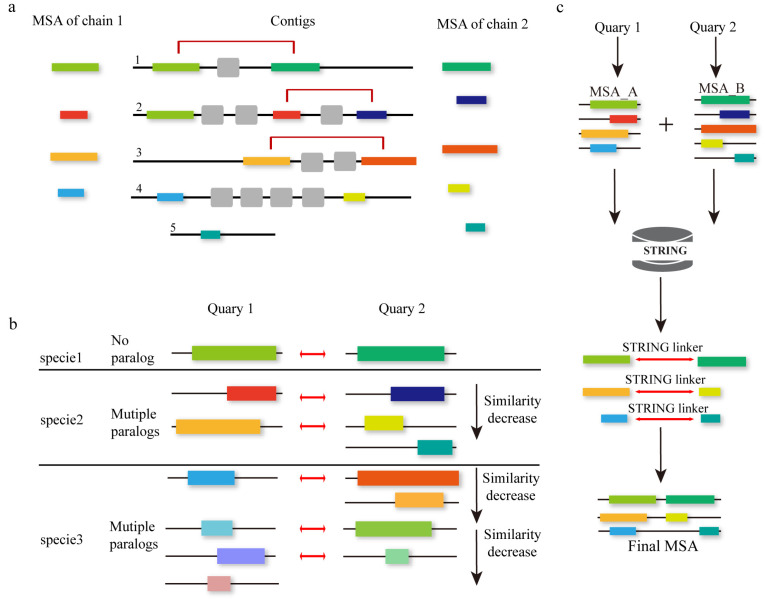
Three MSA concatenation methods for protein complex. (**a**) Genomic distance-based approaches. (**b**) Phylogeny-based approaches. (**c**) Protein–protein interactions databases-based approaches.

Methods like EVcomplex [23] and Gremlin–Complex [91] first construct monomer MSAs using external programs like JackHMMER and HHblits, followed by concatenating the generated MSAs. The MSA concatenation is primarily based on genomic distance distributions (Figure 9a), with the built MSAs filtered using a specified threshold under the assumption that proteins closer on the genome, such as those within the same operon, are more likely to interact. Finally, residue-level protein contact prediction is achieved through the pseudo-likelihood method based on the MSA of the protein complex.

#### 2.2.2. Phylogeny-Based Approaches

In 2018, Hong Zeng et al. proposed ComplexContact [24], an innovative program that built higher-quality MSAs by combining two different MSA concatenation methods and employing a deep learning model to predict inter-protein and residue-residue contacts without using any structural templates. The MSA construction process involves two stages: first, HHblits is used to construct monomer MSAs for two protein subunits respectively; then, these two MSAs are concatenated using two different methods. One method is based on genomic distance, which is similar to the MSA construction theory of EVcomplex and Gremlin–Complex, which suggests that co-regulated genes are often co-located on the genome into operons. The other one is the phylogeny method that categorizes proteins within each MSA by species or subspecies and ranks them according to their sequence similarity to the respective query proteins (Figure 9b). Proteins with identical ranks across the MSAs are then aligned together. For eukaryotes, the phylogeny-based method outperforms the genomic distance method. For prokaryotes, the opposite is true. Therefore, combining these two MSA concatenation methods yields superior results. However, both methods may perform poorly for some protein pairs due to the inability to identify many sequence homologs for their MSAs. The ComplexContact method won the CASP12 competition, accurately predicting inter-protein and residue-residue contacts without requiring extensive sequence homologs, by effectively utilizing co-evolutionary information, sequence features, and contact occurrence patterns. Later, AlphaFold2-Multimer [92] also adopted similar ideas in their multimer MSA construction step.

#### 2.2.3. Protein–Protein Interactions Databases-Based Approaches

In response to the growing demand for knowledge about protein–protein interactions, numerous databases have emerged, with STRING [93] standing out as a prominent example, housing both known and predicted protein interactions. These databases offer a valuable resource for improving the quality of MSA for protein complexes, exemplified by the innovative cpxDeepMSA [25] method. In 2022, Liu et al. introduced cpxDeepMSA, which builds upon the foundation of DeepMSA, employing three distinct strategies for homology detection cpxDeepMSA constructs MSA for protein complexes via three stages. The first stage employs HHblits to search the UniClust30 [86] database for each protein monomer and build MSAs for each monomer. Subsequently, these MSAs undergo comparison within the genome database (ENA) [94]. Ultimately, based on gene distance, the final complex MSA is identified. In stage 2, sequences of each monomer MSA obtained in stage 1 are compared with the taxonomy database from NCBI [95]. The sequences in each monomer MSA are divided by species and ranked by the sequence similarity. The complex MSA is the combination of several monomer MSAs from the same species family. The final stage harnesses the protein–protein interaction information of the STRING linker (Figure 9c). Using HHblits, each monomeric protein is searched against the STRING database, producing the corresponding MSAs. These MSAs are then integrated to form complex MSAs if they are identified as potential interactions based on STRING linker information. The complex MSAs obtained from the three stages undergo scoring and sorting using the Nf of the protein complex MSA. The final complex MSA serves as input for the removal of redundant sequences. In conclusion, cpxDeepMSA represents a robust approach to MSA refinement by leveraging diverse homology detection strategies and tapping into protein interaction databases. Through systematic integration of information from multiple sources, this method holds promise for advancing our understanding of protein complexes and their interactions.

#### 2.2.4. Protein Language Models-Based Approaches

In spite of the above MSA construction methods that are all hand-crafted approaches and merely have effects on the specific domains, new multimer MSA construction methods leveraging PLM [31,32,34] (the details of PLMs can be found in Section 2.4) were first introduced by Bo et al. in 2023, called ESMpair [26]. Different from previous methods, ESMpair does not rely on genetic distance or species information. Instead, it utilizes co-evolution scores learned by PLMs to link MSAs, achieving automation and providing benefits to downstream applications such as contact prediction, remote homology detection, and mutation effect prediction. ESMpair leverages column-wise attention scores from MSA Transformer [31] to identify and pair monomer MSAs. With an input of a pair of query sequences, ESMpair first searches the UniProt [96] database with JackHMMER [82] to generate the MSA for each query sequence. Then, the sequences of the same taxonomy are grouped into the same cluster. MSA Transformer is utilized to calculate the column attention score between each sequence homolog of MSA with the query sequence. Two sequence homologs of the same taxonomy group with similar attention scores from the two query sequences are matched to the generated multimer MSA.

Later, Umberto et al. introduced DiffPALM [97], which utilizes an MSA Transformer for differentiable multimer MSA construction by predicting paralog matchings. Although DiffPALM, like ESMpair, utilizes the MSA Transformer PLM, its method for pairing interacting protein sequences differs from ESMpair’s approach of matching sequences based on similar attention scores. Given that the MSA Transformer inherently captures inter-chain co-evolutionary signals, the MLM loss decreases as the scores for correctly matched sequences increase. Thus, DiffPALM uses masked language modeling (MLM) loss as a co-evolutionary score and seeks pairings that minimize this loss.

#### 2.2.5. Hybrid Approaches for Protein Complex’s MSA

Hybrid approaches, as a strategy that integrates multiple techniques and methods, are fundamentally designed to provide accurate alignments for protein complex structure prediction.

**Figure 10:**
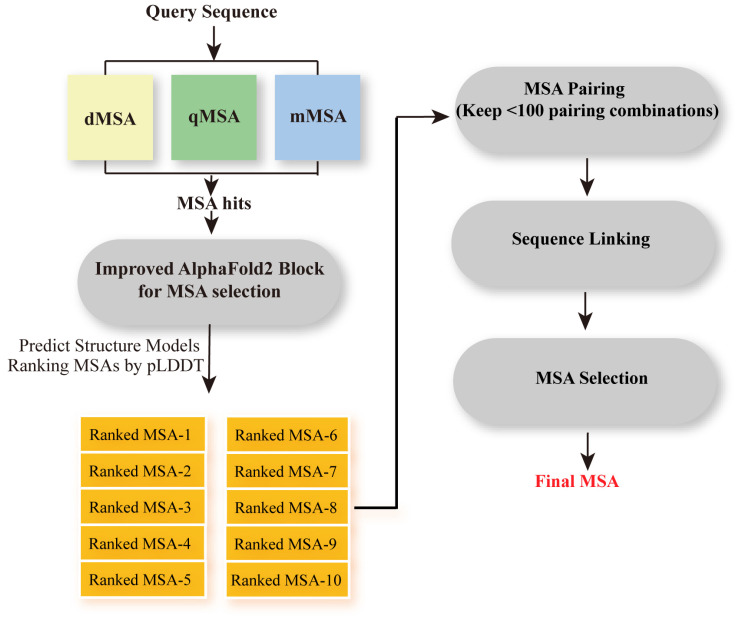
Pipeline of DeepMSA2-Multimer. First, DeepMSA2-Monomer generates monomer MSAs for each chain. Second, MSA pairing is performed for homomeric complexes. Third, paired MSAs are combined. Fourth, the optimal multimer MSA is selected based on Nf and pLDDT.

DeepMSA2-Multimer (Figure 10) is a method for constructing MSAs for protein complexes using homology relationships among component chains [21]. Rather than introducing a novel sequence-linking approach, it provides a pipeline for selecting the most optimal MSA from several alternatives to facilitate subsequent pairwise linking. Here is a streamlined overview of the process. The first step is to generate the monomer MSA of each chain included in the complex using DeepMSA2-Monomer, as we described in Section 2.1, retaining up to 10 MSAs with the highest pLDDT scores per chain to capture diverse alignments. Step two is MSA Pairing. For homomeric complexes (identical component chains), we repeat monomer MSAs *n* times (*n* = number of chains). For heteromeric complexes (different component chains), we select the top *M* MSAs for each monomer chain and construct up to *M^N^* paired MSAs, ensuring *M^N^* ≤ 100. In the third step, paired monomer MSAs are concatenated to form multimer MSAs. Sequences of each monomer MSA are grouped based on UniProt annotated species. Within each group, sequences are sorted by sequence identity with the query sequence. Top sequences from the same species are then connected side by side. Then, we fill in gaps where sequences are missing and add unpaired sequences below. This step is specific to heteromers. In step four, the optimal multimer MSA is selected based on depth (Nf) and folding scores (pLDDT) from monomer MSAs. This step primarily applies to heteromeric complexes; for homomeric complexes, all 10 concatenated MSAs are retained. In summary, DeepMSA2-Multimer systematically creates, pairs, connects, and selects MSAs to provide accurate alignments for protein complex structure prediction, enhancing insights into protein interactions. Moreover, with the help of DeepMSA2-Multimer, DMFold-Mulimer outperformed AlphaFold2-Multimer in protein complex structure prediction in CASP15.

Meanwhile, Liu et al. introduced MULTICOM [98], featuring an enhanced multimer MSA construction module designed to optimize the input for AlphaFold2-Multimer, to achieve more accurate protein complex structure predictions. MULTICOM first extracts monomer sequences from multimer targets. Then, it uses sequences alignment tools including HHblits, JackHMMER, MMseqs2, and their in-house implementation of DeepMSA to search against UniClust30 [86], UniRef30, UniRef90 [99], UniProt [100], the IMG database [101], and the metagenome sequence database to build MSAs for each monomer sequence. Secondly, monomer MSAs are concatenated. For hetero-multimers, the alignments in the MSAs of the subunits are concatenated using the potential protein–protein interaction information extracted from multiple sources to construct MSA-paired (the paired MSA that may encode the coevolutionary information between the subunits), including species annotations, UniProt accession numbers, protein–protein interactions in the STRING database, and complex structures in the Protein Data Bank (PDB). This process generates thirteen types of MSA-paired. MSA-unpaired is padded beneath MSA-paired to minimize the loss of evolutionary information during monomer structure prediction. For homo-multimers, MULTICOM uses AlphaFold2-Multimer’s default method to create MSA-paired from various databases. Custom methods pair only subunits with the same species annotation or PDB code, others are paired with gaps. Only MSA-paired is used in structure generation, while MSA-unpaired is ignored. Meanwhile, structural templates are retrieved by searching the template database. Combining input of diverse MSAs and structural templates and AlphaFold2-Multimer confidence score with the complementary pairwise prediction similarity score to rank predictions. Enhancing the diversity of MSAs and structural templates elevated the accuracy of the top models predicted by AlphaFold2, which contributed to MULTICOM’s outstanding performance in CASP15 [102].

### 2.3. Multiple Sequence Alignment for RNA

A profound understanding of RNA structure and function is crucial for addressing a range of biological questions, with the extraction of evolutionary and co-evolutionary information embedded within RNA through MSA serving as a critical entry point. The previously mentioned protein MSA construction tools do not account for base-pairing relationships within RNA secondary structures (rSS). Consequently, in recent years, numerous specialized tools for constructing RNA MSAs have been developed [28,29,103,104] to produce more accurate and biologically relevant alignments. These tools encompass sequence-based approaches, HMM-based approaches, CM-based approaches, and hybrid approaches.

#### 2.3.1. Sequence-Based Approaches for RNA’s MSA

FASTN [57] is a subroutine within the FASTA suite that utilizes the same core algorithmic principles as FASTA, while BLASTn [27] is a tool within the BLAST suite, based on the same core algorithmic principles of BLAST. Both FASTN and BLASTn are specialized for rapid alignment of nucleic acids (DNA or RNA), employing heuristic algorithms for efficient searching. They take one or more nucleic acid query sequences as input and produce alignment results against nucleic acid sequences in the database. Both tools demonstrate robust performances, particularly with large datasets.

#### 2.3.2. HMM-Based Approaches for RNA’s MSA

nhmmer [28] is a DNA/RNA sequence comparison tool based on the framework of HMMER. Similar to the core concept of HMMER that was previously introduced, it enables sequence alignment by allowing position-specific residue and gap scoring based on the query profile, and utilizes the more robust Forward/Backward HMM algorithm to calculate homology signals. The key distinction of nhmmer lies in its focus on searching nucleotide sequence databases (NT), with particular attention to chromosome-length target sequences and the extreme composition biases frequently encountered in genomic DNA. Specifically, nhmmer outputs a ranked list of hits with the most significant matches to the query, where each hit represents a local alignment of the profile to a subsequence of a target database sequence, rather than to a full sequence in the target database. Furthermore, nhmmer employs a series of acceleration filters, refined from HMMER, to enhance performance. The initial “single segment ungapped Viterbi” approach trades some precision for rapid scanning of the target sequence, with high-scoring regions subjected to a subsequent full-gapped Viterbi alignment. Candidate alignments filtered through the initial two stages are subsequently subjected to the full rigor of Forward/Backward alignment, a process that incorporates correction for compositional biases. The aforementioned improvements endow the algorithm with the dual advantages of high sensitivity and reduced computational time.

#### 2.3.3. Covariance Model-Based Approaches

When searching for homologous RNAs in sequence databases, incorporating consensus secondary structure annotations can optimize the results. Stochastic context-free grammars (SCFGs) provide a natural statistical framework for integrating sequence and secondary structure conservation information into a unified scoring system. The Infernal program [29], proposed by Eddy et al. in 2009, utilizes covariance model (CM), a specific form of SCFGs, to construct consensus RNA profiles for either single RNA sequences or MSAs with consensus secondary structure annotation, facilitating RNA database searches and MSAs. CMs are closely related to profile HMMs, commonly used in protein sequence analysis, but are more complex. Both CMs and profile HMMs capture conservation information at each alignment column; however, while positions are treated independently in profile HMMs, base-paired positions in CMs are interdependent. Specifically, CMs consist of many states of these seven basic types, each with its own unique emission and transition probability distributions, as well as a set of permissible transitions. Ultimately, CMs assign position-specific scores for the four possible residues at single-stranded positions, the 16 possible base pairs at paired positions, and insertions and deletions.

**Figure 11:**
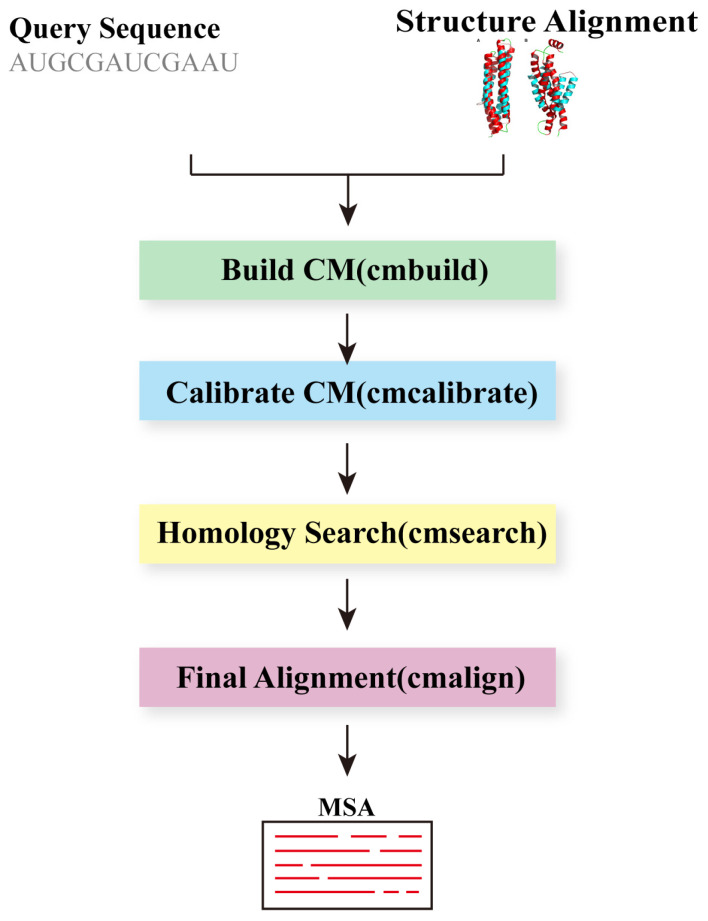
The Workflow of Infernal. It holds a critical position in RNA MSA due to its ability to incorporate rSS information into the alignment process, enabling precise homology search and structure prediction. The workflow begins with the input of a query RNA sequence and structure alignment, followed by the construction and calibration of a CM, the search for homologous RNA sequences in the database, and the implementation of precise alignments, ultimately producing an MSA output. The introduction of the CM as a core concept in RNA MSA significantly enhances Infernal ‘s impact in the field.

The primary steps for obtaining an MSA with Infernal are as follows (Figure 11): first, ‘cmbuild’ is used to build a CM from a structural alignment. Next, we calibrate the CM for homology search with ‘cmcalibrate’. Then, Infernal employs ‘cmsearch’ to search for putative homologs in the database. Finally, these identified homologs are aligned to a CM using ‘cmalign’. It is noteworthy that in the ‘cmcalibrate’ step, the application of a two-stage filtering technique greatly reduces the computational time during the search phase without significantly compromising sensitivity. The first filtering technique employed is the HMM filtering, with thresholds configured by ‘cmcalibrate’. Subsequently, the query-dependent banded (QDB) CYK maximum likelihood search algorithm is utilized as the second filter, with relatively tight bands set. The new version of Infernal [105], released in 2013, introduced several improvements. Notably, the search speed was further enhanced due to the integration of HMMER3’s accelerated filtering algorithms and constrained CM alignment algorithms. The introduction of the ‘cmscan’ program allows users to identify which structural RNAs are present in a collection of sequences. Additional enhancements include more precise handling of truncated RNAs.

#### 2.3.4. Hybrid Approaches for RNA’s MSA

Furthermore, RNAcmap, RNAlien, and rMSA have been proposed to implement fully automated pipelines, building on the classic algorithms BLASTn, nhmmer, and Infernal.

RNAlien [30] employs an iterative search strategy, MSA, and CM construction, aiming to automatically search and generate all homologous sequences starting from a single sequence, including more difficult-to-detect remote homologs. The overall process of RNAlien is relatively straightforward. Initially, a BLASTn search is performed, followed by secondary structure consensus filtering. This step helps identify sequence-similar candidates within the close taxonomic neighborhood of the input sequence. Subsequently, initial structural alignment and CM are constructed using tools such as ‘cmbuild’ and ‘cmcalibrate’. Finally, BLASTn continues to expand the search to more distantly related species, with the CM used to decide whether to include new candidate sequences in the initial set. After all species have been explored, the generated CM, structural alignment, and all collected homologous sequences are returned. In summary, this method uses iterative searching and structural conservation filtering to emphasize the collection of distant homologous members, thereby enhancing the sensitivity and diversity of the model.

RNAcmap [106] is a fully automatic pipeline that enables evolutionary coupling analysis for any RNA sequence. Within its pipeline, the homology search step generates MSA. RNAcmap initially conducts a homology search in the NT using BLASTn [27] to obtain the initial MSA. Simultaneously, RNAcmap employs either the folding-based algorithm RNAfold [107] or the deep learning method SPOT-RNA [108] for secondary structure prediction. Afterward, the initial MSA and the predicted consensus secondary structure are input into Infernal ‘s [105] ‘cmbuild’ tool to construct a CM. Following calibration with ‘cmcalibrate’, a second round of searching is performed in the NT where the E-value for ‘cmsearch’ is set to 10 [109] to encompass more homologs with lower sequence identity. Finally, the aligned homologous sequences are obtained for subsequent evolutionary coupling analysis. The efficacy of RNAcmap is on par with that of manually curated Rfam alignments. Significantly, its performance demonstrates robustness across sequences that fall outside Rfam families, as well as pseudoknot RNAs and non-redundant RNA sets.

rMSA [110] is a hierarchical pipeline designed for the search and alignment of RNA homologs for a target RNA, significantly improving the prediction of rSS and contacts. This algorithm employs a novel five-stage hierarchical sequence search strategy which avoids the excessive inclusion of irrelevant sequences. In Stage 1, BLASTn aligns the target RNA against RNAcentral and NT databases, producing initial hits. nhmmer40 realigns these hits to form the initial alignment. This alignment is then converted to a CM using Infernal. The cmsearch program of Infernal employs this CM to perform a profile-sequence search through BLAST hits, resulting in the Stage 1 MSA. In stages 2 and 3, the CM generated in the first stage is utilized to search the RNAcentral and NT, respectively. The raw ‘cmsearch’ hits from these stages are merged with hits from the preceding stage and realigned to produce the Stage 2 and 3 MSAs by ‘cmsearch’. During stages 4 and 5, the target sequence is individually searched against the RNAcentral and NT using BLASTn. The resulting BLAST MSAs are converted into CM 2 and CM 3, respectively, compensating for any potential omissions in the nhmmer realignment stages of the first three stages. Consistently, the same predicted secondary structure from RNAfold is employed in building CMs during stages 1, 4, and 5. CM 2 and CM 3 are then utilized by ‘cmsearch’ to search through sequences gathered from the preceding three stages, yielding the Stage 4 and 5 MSAs, respectively. At each stage, a length-normalized number of Nf is calculated, and the process proceeds to the next stage only when Nf < 128 [81], thereby avoiding unnecessary construction of large MSAs. For the final MSA selection, rMSA constructs an MSA score based on PLMC [111] covariance. The MSA score measures the consistency between single-sequence-based and MSA-based rSS predictions. MSAs with greater diversity and more homologous sequences are expected to yield stronger covariance signals and more sensitive predictions for base pairing and contacts. Through these processes, the MSA construction method of rMSA based on sequence-sequence and profile-sequence ensures sufficient depth and coverage, consistently and significantly improving predictions of rSS and contacts compared to existing RNA MSA generation programs, while avoiding redundant or irrelevant large-scale MSAs.

### 2.4. Alternative for MSA in Application Tasks, Protein Language Model

**Figure 12:**
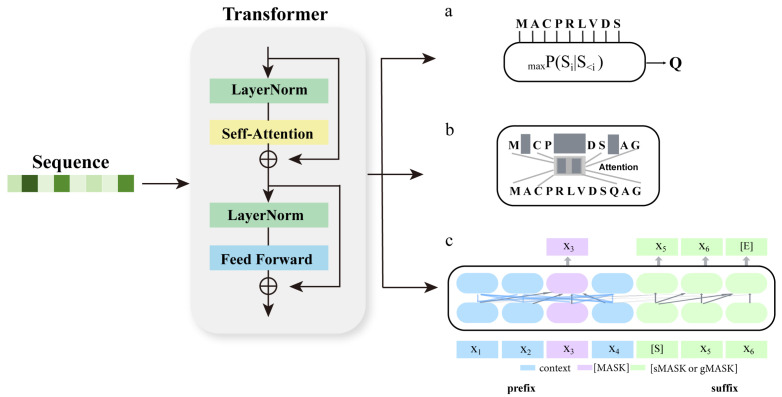
The schematic diagram illustrates the core modules and training objectives of PLMs. (**a**) PLMs based on autoencoder frameworks tend to learn protein representations for downstream task analysis, particularly in tertiary structure prediction. (**b**) PLMs utilizing autoregressive frameworks are inclined to generate proteins in novel sequence spaces. (**c**) General models like T5 and GLM are capable of performing both tasks.

Breakthroughs in protein design and structure prediction fields have been achieved by leveraging the rich biological information from MSAs. However, the construction of MSAs is constrained by various factors: the process is time-intensive [17]; retrieval schemes rely on inefficient manual design; and not all proteins can access a plentiful and diverse collection of high-quality homologous sequences [34]. These limitations have somewhat hindered the development of protein-related fields heavily dependent on MSAs. At present, a highly efficient MSA alternative methodology in application tasks involves training large-scale PLMs based on various training objectives, such as autoencoding (Figure 12a), autoregressive (Figure 12b), and other types of approaches (Figure 12c), to extract a wide range of features. These features capture evolutionary and co-evolutionary information, making PLMs a viable alternative to MSAs in tasks such as function prediction, contact prediction, tertiary structure prediction, and protein design. Its effectiveness has been demonstrated to be comparable to state-of-the-art MSA-based methods.

#### 2.4.1. PLMs with MSA as Input

The MSA Transformer (ESM-MSA-1b) amalgamates a methodology centered on extracting insights from the covariance among mutations across columns within MSAs, e.g., Potts model. Through unsupervised learning, MSA Transformer trains a deep Transformer [31] model capable of handling multi-sequence alignment forms of input. Specifically, by employing accelerated and optimized HHblits in HH-suite3 [112] to query the UniClust30 [86] database, MSAs were constructed for each sequence contained within the UniRef50 [99] database. The training dataset encompasses 26 million MSAs, averaging 1192 sequences per alignment. The pre-trained model is endowed with 100 million parameters, consisting of 12 layers, a 768-dimensional embedding size, and 12 attention heads. To effectively handle multi-sequence inputs and fully leverage the matrix structural features inherent in MSA while mitigating excessive memory requirements, the model adopts the axial attention approach [113]. The row attention modules and column attention modules are arranged alternately, and a variant called Tied Row Attention is proposed, wherein a single attention map is shared among rows. This operation not only reduces computational costs but also imposes constraints on each sequence within an MSA to possess similar structures. The pretraining strategy involves randomly and uniformly masking tokens on the MSA or masking entire columns of the MSA, followed by predicting the identities of the masked tokens.

MSA2Prot [114] also takes MSA as input. However, unlike the MSA Transformer, this method incorporates an additional decoder that explicitly autoregressively models sequence probabilities, thereby enabling sequence generation. The encoder–decoder model was trained on the full set of 10,593 Pfam family alignments. The encoder is structured as a stack of transformer layers with axial attention applied to both the rows and columns of the MSA, while the decoder layers consist of causal self-attention, cross-attention to the MSA representations, and fully connected layers with layer normalization and residual connections for each block. The pre-trained model uses 6 encoder and decoder layers, each with a hidden dimension of 768, where the MSA encoder employs 12 attention heads and the decoder utilizes 8 attention heads. The model parameters are optimized by minimizing the negative log-likelihood of the target sequence conditioned on its family MSAs.

#### 2.4.2. Autoencoding PLMs with Single-Sequence Input

The ESM series represents a typical example of PLMs based on autoencoder architectures. ESM-1b [32] trains a deep Transformer [88] architecture network model via unsupervised learning to acquire amino acid-level representations imbued with contextual information. Specifically, the approach utilizes the encoder of the Transformer, characterized by a sequence of blocks where self-attention layers and feed-forward connection layers alternate to process the input. The model is trained on a dataset consisting of 250 million protein sequences and 86 billion amino acids sourced from the UniRef50 database, employing MLM objectives. This results in a pre-trained Transformer model with approximately 650 million parameters across 33 layers. Later, the MSA Transformer (ESM-MSA-1b) [31] method was proposed, which, in contrast to the single-sequence input approach, utilizes MSA as input. A detailed discussion of this method can be found in Section 2.4.1. Subsequently, ESM-2 [115] continues the BERT-style Transformer architecture and MLM strategy of ESM-1b. It was trained on approximately 65 million non-redundant sequences from UR50/D, employing a range of models with different parameter sizes. The largest model in this series contains 15 billion parameters, with 48 layers, an embedding dimension of 5120, and 40 attention heads. Additionally, Rotary Position Embedding (RoPE) was used in place of the learned sinusoidal encoding employed in ESM-1b.

The ProtTrans [34] series leverages language models and transfer learning in protein research, pretraining six distinct models, three of which are based on autoencoding architectures (ProtBert [116], ProtAlbert [117], ProtElectra [118]). Specifically, ProtBert trained a Bert [116] model with 420 million parameters on BFD100 and UniRef100 datasets and enhanced the original Bert by increasing the number of layers. Bert is the first bidirectional language model used to reconstruct masks and is considered to be the standard for NLP transfer learning. ProtAlbert trained an Albert [117] model with 224 million parameters on UniRef100. Through factorization embedding parameterization and cross-layer parameter sharing, the number of parameters is reduced compared to those of the original Bert, while the number of attention heads is increased. ProtElectra trained an Electra [118] model with 420 million parameters on UniRef100, utilizing a generator to produce reasonable alternative tokens and a discriminator to identify replaced tokens, employing adversarial training principles to enhance efficiency and performance.

The representations obtained from PLMs can be applied to a variety of downstream protein understanding tasks, including remote homolog detection, secondary structure prediction, residue–residue contact prediction, mutation effect prediction, subcellular localization at the protein level, and the prediction of membrane proteins versus soluble proteins, among others. Their performance can rival that of advanced MSA-based methods. Notably, ESMFold, based on ESM-2, has achieved high-resolution atomic-level protein structure prediction for the first time using a PLM instead of MSA. These findings highlight the potential of PLMs as viable alternatives to traditional MSA methods in application tasks.

Therefore, enhancing the quality of embeddings produced by PLMs is a critical focus for future research. For instance, the previously discussed MSA Transformer improves model performance by integrating prior information from MSA during training. Additionally, ProteinBERT leverages functional annotations as supplementary information, while Saprot incorporates structural data. ProteinBERT [119] is a denoising autoencoder inspired by the Bert architecture, which performs dual reconstruction during pretraining on masked amino acids and GO functional annotations of proteins. The two parallel pathways independently process sequences and functional annotations, obtaining local and global representations respectively. The dual training tasks enable high-quality unsupervised learning even with a smaller parameter count, showcasing performance comparable to larger-scale PLMs in tasks such as secondary structure, remote homology, fluorescence, and stability prediction. Saprot [120] represents the inaugural universal PLM developed based on an extensive collection of AlphaFold2-predicted structures, capable of extracting both sequence co-evolutionary information and structural information for a diverse array of downstream tasks. This model leverages Foldseek [40], which is based on VQ-VAE [121], to convert protein structures into structural tokens for each amino acid position, thereby representing different 3D interaction (3Di) states. The 3Di alphabet describes tertiary contacts in 3D space by approximating the local backbone conformations of each residue *i* and its nearest neighbor *j* using 20 discrete states. By combining these structural tokens with amino acid-type tokens, a comprehensive SA vocabulary comprising 441 unique tokens is generated. The architecture and parameter size of the Saprot model align with the 650M version of ESM2, which will be described in detail later, with a straightforward substitution of sequence tokens with SA-tokens. Pre-training is conducted on a dataset encompassing approximately 40 million protein structures, utilizing a BERT-style MLM objective. A distinctive feature of Saprot is its approach of randomly masking either the structural token or the sequence token for a given amino acid, but never both simultaneously, which helps mitigate erroneous optimization directions that could result from inaccurate SA-token outputs by Foldseek. Saprot exhibits superior zero-shot mutation effect prediction capabilities compared to ESM-2, structure-based models such as MIF-ST [122] and ESM-IF, as well as MSA-based models like Tranception L, MSA Transformer, and EVE [123]. Additionally, Saprot demonstrates exceptional performance across eight supervised prediction tasks, including Thermostability, HumanPPI, and Metal Ion Binding, underscoring its robust and versatile representational capacity. However, the current body of work on Saprot does not investigate its potential for structure prediction based on single sequences.

As the scale of training data and model parameters for PLMs continues to expand, the representational capacity of the generated sequence embeddings is progressively enhanced. In previous studies, the structural information captured by PLM representations from single-sequence inputs has remained confined to relatively low-resolution levels, particularly regarding secondary or tertiary structures. However, more recent PLMs, like AminoBERT [124] and OmegaPLM [125], encapsulate sufficient structural information in their representations, enabling the performance of subsequent structure prediction tasks based on these models to rival that of MSA-based methods. AminoBERT was trained on an extensive corpus of approximately 250 million natural protein sequences from the UniParc database [126], utilizing a Transformer architecture characterized by 12 attention heads and an output sequence representation dimension of 3072. To enhance the model’s capacity to capture global sequence information, two novel training objectives were introduced: firstly, with a probability of 0.7, the model masks 2–8 consecutive residues and subsequently predicts their true identities; secondly, with a probability of 0.3, the model alters the order of adjacent sequence fragments through the chunk permutation technique and discerns whether the sequence has been modified. OmegaPLM, trained on the Uniref50 dataset with 670 million parameters, differs from traditional PLMs by employing 66 GAU layers instead of self-attention layers and MLPs, allowing for lower memory requirements and faster convergence. It incorporates Pre-LayerNorm and uses RoPE similar to ESM-2. Unlike other PLMs, OmegaPLM ‘s training objectives align with ESM-1b’s BERT masking, and it additionally integrates an optimized spanBERT-like [127] loss and Sequential masking. To enhance the model’s focus on long-range amino acid relationships, Focal Loss [128] is utilized.

#### 2.4.3. Autoregressive PLM with Single-Sequence Input

Another class of PLMs, based on autoregressive frameworks, similarly captures the consistent underlying dependencies between protein sequences, primarily excelling in tasks related to sequence generation.

Among the six models in the ProtTrans series, ProtTXL and ProtXLNet are based on autoregressive architectures. Specifically, ProtTXL trained Transformer-XL [129] models with 409 million and 562 million parameters on the UniRef100 and BFD-100 datasets, respectively. The BFD integrates proteins translated from multiple metagenomic sequencing projects and UniProt, constituting the largest protein sequence collection at the time. The advantage of Transformer-XL lies in its variant of the transformer architecture introducing a segment-level recurrence mechanism, allowing it to handle protein fragments of arbitrary lengths, partially alleviating the constraints on long sequences. ProtXLNet trained XLNet [130] models with 409 million parameters on the UniRef100 database, employing a similar memory mechanism to handle sequences of arbitrary lengths, further optimizing Transformer-XL by addressing its unidirectional context limitation and enabling the collection of bidirectional contextual information.

The ProGen [131], with a parameter scale of 1.2 billion, was trained on a dataset comprising 280 million non-redundant protein sequences along with their corresponding control tags sourced from UniParc [126], UniprotKB [132], Pfam [133], and NCBI taxonomic information [95]. The model is a 36-layer transformer network, with each layer comprising 8 self-attention heads. The control tags were divided into two categories: keyword tags and taxonomic tags, which covered terms related to cellular components, biological processes, molecular functions, and taxonomy spanning across eight standard taxonomic ranks from NCBI. A key advantage of ProGen lies in its ability to leverage specified control tags to guide sequence generation, enabling precise control over protein family, biological process, and molecular function properties, significantly enhancing the diversity of protein sequences across different families.

Compared to ProGen, ProGen2 [134] was trained on a broader dataset of 1 billion protein sequences from genomic, metagenomic, and immune repertoire databases, utilizing models with parameter sizes ranging from 151 million to 6.4 billion. The model architecture follows a standard left-to-right autoregressive transformer decoder with causal masking, employing RoPE and executing self-attention and feed-forward circuits in parallel to optimize communication overhead. ProGen2 achieves state-of-the-art performance in generating sequences and accurately predicting protein fitness without the need for additional fine-tuning, effectively capturing the evolutionary sequence distributions.

The model architecture of ProtGPT2 [33] adopts HuggingFace’s autoregressive GPT2-large Transformer [135], with a parameter scale of 738 million. The model consists of 36 layers with a dimensionality of 1280, utilizing the original dot-scaled self-attention mechanism. The token sequences obtained by applying the Byte Pair Encoding (BPE) strategy to the 44.88 million sequences in the UniRef50 dataset were used as input training data. It can generate new protein sequences consistent with the stability, kinetic properties, and disorder propensity of natural proteins within unknown sequence spaces, and fine-tuning can enrich the diversity of sequences within specified protein families.

RITA [136] trains an autoregressive GPT-3 model with a parameter scale of 1.2 billion, utilizing Prompt Tuning to generate controllable protein sequences.

Tranception [137], another autoregressive-based model, shows significant promise in the field of protein design. Distinguished from preceding models, it integrates techniques from Primer and Inception, giving rise to a novel Tranception attention mechanism. This mechanism focuses on extracting information from contiguous subsequences of size *k*-mer, and during inference, it combines with a homologous sequence retrieval module. These advancements enable Tranception to achieve state-of-the-art results in protein fitness prediction tasks and to handle indels, a capability lacking in ESM-1v [138] and MSA-Transformer.

#### 2.4.4. Other Types of PLMs

The two mainstream pre-trained PLMs frameworks discussed earlier each possess distinct advantages and limitations. Autoencoding models leverage denoising objectives to learn bidirectional context encoders, rendering them well-suited for comprehension tasks but not directly applicable to sequence generation. Autoregressive models, which learn language modeling in a left-to-right fashion, are advantageous for generating extended sequences and few-shot learning, though they fall short in capturing bidirectional contextual dependencies. Building on these foundations, ProtT5 [34] and xTrimoPGLM [139] have been introduced. These models employ architectures distinct from those previously mentioned, facilitating a more profound extraction and assimilation of protein sequence features.

In the ProtTrans [34] series, ProtT5-XL and ProtT5-XXL are T5 [140] models pre-trained on the UniRef50 and BFD100 databases, with parameter sizes of 3 billion and 11 billion, respectively. The T5 models are designed to transform various protein-related tasks into a text-to-text format, thereby offering a universal model framework. A key feature of these models is their simultaneous use of both the encoder and decoder components of the Transformer architecture, the encoder employs bidirectional attention, while the decoder utilizes unidirectional attention, with cross-attention mechanisms connecting the two, which mitigates the limitations associated with using only one of these components.

XTrimoPGLM [139] is a unified PLM based on the General Language Model (GLM), aiming to integrate objectives from different frameworks. The GLM represents a more efficient general language model compared to T5, introducing two key innovations and improvements. Firstly, it employs an autoregressive blank-filling training objective, which differs from the MLM task. In GLM, sequence segments are replaced with a ‘MASK’ symbol, and each masked segment is predicted autoregressively until the prediction token is an end-of-sequence marker. This approach not only requires the model to predict the correct token but also to enhance its ability to correctly predict the length of the masked segments. Secondly, GLM utilizes a 2D positional encoding scheme, where each token is encoded with two positional IDs: the first dimension represents the position of the corrupted text within the original sequence, and the second dimension records the position within the masked segment area. The parameter scale of xTrimoPGLM reaches a record-breaking 100 billion for the first time, resulting in a significant improvement in handling multiple protein understanding tasks. Moreover, it can generate new sequences with functional structures distinct from natural proteins in larger sequence spaces, further advancing the field of protein research.

## 3. Discussion

Sequence alignment, a cornerstone task in the analysis of biological sequences, has consistently maintained a pivotal role within bioinformatics. By searching for homologous sequences in large-scale databases and constructing MSAs, the intricate relationships between diverse sequences can be extensively explored. This approach holds significant value for structure prediction, functional analysis, and evolutionary studies, offering profound insights into the molecular mechanisms of biological systems. Moreover, it drives progress in drug discovery, the investigation of disease mechanisms, and research on environmental adaptability.

Approaches to constructing MSAs for protein monomers encompass sequence-based, HMM-based, *k*-mer-based, hybrid, and deep learning-based methods. The strengths and limitations of the various methods are comprehensively outlined in Table 2. When target sequence similarity exceeds 30%, homology can be comprehensively detected using sequence-to-sequence and sequence-to-profile approaches. Conversely, when sequence similarity drops below 30%, methods predicated on profile HMM and HMM-to-HMM alignments effectively address the limitations of the former techniques, thereby serving as superior tools. With the rapid advancements in sequencing technologies, biological sequence data has grown exponentially, leading to the development of *k*-mer-based methods to meet the increasing demands for accuracy, automation, and sensitivity in sequence alignment. Additionally, hybrid strategies, which combine various advanced search methods and alignment techniques, have been introduced to enable automated and rapid searches across large-scale datasets and metagenomes. However, when evolutionary relationships are highly divergent, the lack of sequence conservation exacerbates the challenge of identifying remote homologies. Given that structural divergence occurs more slowly than sequence divergence, some methods incorporate structural similarity for detection. It is important to note, however, that while homologous proteins are likely to exhibit high structural similarity, proteins with high structural similarity are not necessarily homologous. In addition to structural information, PLMs, due to their rich embedded biological information, have been utilized as indirect supplementary data for homologous protein searches. This allows them to achieve speeds comparable to state-of-the-art sequence-based methods and sensitivity on par with cutting-edge structure-based approaches. Overall, the methods for constructing monomeric protein MSA are well-established. However, most algorithms currently do not account for the alignment of multidomain proteins, which remains a challenging issue due to the difficulty in defining domain boundaries. HMMER, through the stochastic traceback clustering algorithm, effectively identifies and parses multidomain protein sequences, recognizing each domain and aligning it with the corresponding model without confusing or overlapping the domains. The DCTdomain [141] method, proposed by Benjamin Giovanni Iovino et al., leverages protein sequence embeddings and contact map predictions from ESM-2 to identify domains. It then applies discrete cosine transformation (DCT) to generate domain-based embeddings (DCT fingerprints), which facilitate the fast and accurate detection of protein similarity. On the other hand, the rapidly growing scale of metagenomic databases has made sequence searching increasingly challenging. Therefore, reducing the time required for this process may be a key future development for protein monomer MSA construction methods. One example is the MetaSource model [142], which enhances MSA construction by connecting microbial community data with homologous protein family sequences, thereby speeding up the homologous sequence search and improving the overall alignment process.

As the technology of MSA monomer construction has been developed, the quality of MSA has a greater influence on bioinformatics research. Firstly, protein monomer MSA aids in identifying conserved and variable regions. Conserved regions are segments present across different protein sequences, typically indicative of crucial structural or functional aspects. By aligning multiple protein sequences, scientists can pinpoint these conserved regions and further investigate their roles in protein structure and function. Conversely, variable regions represent differences between sequences, potentially linked to specific functions or evolutionary adaptations. Furthermore, with MSAs, scientists can try to choose the most likely set of mutations that may be potential ligand binding sites for drug targets [9,143]. Secondly, protein monomer MSA is frequently utilized in predicting protein structure. MSA is the primary component to derive local secondary structure features [144,145], residue–residue contacts [146,147,148,149], and homologous structural templates [76,150,151], which are essential for the full-length three-dimensional (3D) structure prediction. With the evolutionary information extracted from MSAs, the accuracy of protein monomer structure prediction has been greatly improved, as shown by AlphaFold2 [4]. In summary, protein monomer MSAs play a crucial role in elucidating protein structure, function, and evolutionary relationships, holding significant relevance across various domains in biology, drug discovery, and life sciences.

The construction of MSAs for protein complexes focuses on selecting and concatenating monomer MSAs for the component chains. This includes strategies based on genome distance, phylogeny, protein interaction databases, PLMs, and hybrid approaches. The advantages and limitations of the various methods are summarized in Table 2. Genome distance-based methods are better suited for prokaryotes, while phylogeny-based methods are more appropriate for eukaryotes. Incorporating protein interaction databases for MSA refinement helps generate more robust results. PLM-based approaches enable highly automated monomer MSA concatenation. Hybrid methods combine various homology detection strategies and monomer MSA concatenation techniques to achieve high-quality, deep, and generalized MSA construction. The methods for constructing MSA for protein complexes are still in the early stages of development. A key challenge is the underutilization of large amounts of unannotated species data in metagenomic databases. Additionally, existing protein interaction databases, such as STRING, have limited data, and there is currently no strong evidence that PLM-based connection methods can provide superior results. In the future, advancements in technologies that enable the acquisition of large-scale, high-quality, and cost-effective protein–protein interaction (PPI) data could significantly benefit the construction of protein complex MSAs.

Similar to monomer MSAs, since multimer MSAs are rich in evolutionary information, they usually shed light on the evolutionary history and divergence of protein complexes which is useful for protein complex contact and distance prediction and protein complex structure prediction [152,153,154,155]. Moreover, multimer MSAs can guide the design of mutagenesis experiments aimed at studying the functional significance of specific residues or domains within protein complexes. By identifying conserved or variable regions, researchers can pinpoint sites for mutagenesis and assess their impact on complex formation and function. From the aspect of protein function annotations, the use of multimer MSAs significantly enhances the accuracy of Gene Ontology (GO) predictions and ligand binding site predictions of protein complexes. In addition to these applications, protein–protein interaction prediction is a significant usage of multimer MSAs. Understanding protein–protein interactions of protein complexes of interest can not only contribute to protein complex structure prediction but also help reveal functional molecular mechanisms and drug target identification.

Incorporating base-pairing relationships to construct high-quality RNA MSAs is essential, paralleling the significance of protein MSAs. Methods for constructing RNA MSAs include sequence-based, HMM-based, CM-based, and hybrid approaches. Table 2 summarizes the strengths and limitations of the various methods. HMM-based methods offer enhanced capability for capturing remote homologous relationships compared to sequence-based methods. CM-based approaches utilize conserved secondary structure features as supplementary information, which is particularly important for identifying functionally similar RNA molecules with significant sequence divergence. The limitations of RNA MSA methods mainly stem from the underutilization of metagenomic sequence databases. Incorporating metagenomic sequences in the future could significantly improve the quality of MSAs.

MSA serves as a pivotal foundation for various RNA structural modeling tasks, including the prediction of rSS, contact maps, and tertiary structures. For example, the Sankoff model [156] and its simplified derivatives such as PMcomp [103], Dynalign [157], consan [104], and LocARNA [158], perform RNA sequence alignment and secondary structure prediction simultaneously. Additionally, methods such as RoseTTAFoldNA [159], DeepFoldRNA [160], and trRosettaRNA [161] rely on the conservation information derived from MSA, integrating deep learning models to extract features and predict the 3D structures of RNA, thereby advancing our understanding of biological phenomena and fostering the development of innovative technologies. Notably, the current methods are limited by their inability to integrate metagenomic sequences, high computational complexity, significant time costs, and heavy reliance on data quality, often necessitating a balance between accuracy and computational resources when handling large-scale RNA sequence data to achieve optimal alignment results. In recent years, RNA language models have emerged to efficiently and accurately analyze RNA sequences, replacing traditional costly experimental techniques. These models perform well across various downstream tasks but encounter challenges in handling 3D structural motifs of RNAs, thereby limiting their ability to elucidate RNA functionality.

MSAs explicitly capture evolutionary and co-evolutionary information of sequences, while PLMs can serve not only as supplementary information for constructing MSAs but also as direct substitutes, enabling implicit and deeper exploration while significantly reducing time costs. Currently, PLMs are primarily categorized into methods based on encoder frameworks, which excel at capturing bidirectional dependencies within context; methods based on autoregressive frameworks, which are proficient in sequence generation through conditional probability modeling; and various other approaches that attempt to integrate both tasks. PLMs can utilize both single-sequence input and MSA for training. The advantage of using MSA as input lies in its ability to allow the model to capture richer sequence relationships and evolutionary patterns, thereby enhancing its capacity to model protein structures and functions across diverse families. Table 2 delineates the advantages and drawbacks of the various frameworks. In protein engineering, language models play a crucial role not only in extracting representations of coevolutionary features for protein understanding tasks, such as structure prediction and functional prediction but also in replacing traditional energy minimization functions [162] or coevolutionary statistical models based on MSA [91,163,164,165] for protein design and generation tasks.

The introduction of the MSA-based end-to-end deep learning approach AlphaFold2 has elevated the performance of 3D structure prediction to a new level. However, the computational cost of constructing MSA is prohibitively high and insufficient to meet the demands of current research. To address this limitation, substantial efforts have been directed toward leveraging representations from PLMs as alternatives to MSAs in application tasks for single-sequence-based 3D structure prediction. Notable approaches include RGN2 [166] based on AminoBERT [124], ESMFold [115] based on ESM-2, and OmegaFold [125] based on OmegaPLM. Although these methods exhibit slightly lower structural prediction accuracy compared to the MSA-based AlphaFold2 [4], they surpass AlphaFold2 in predicting the structures of orphan proteins and de novo-designed proteins, which lack extensive homologous sequences. Moreover, they have achieved remarkable progress in computational efficiency, highlighting their immense potential for practical applications.

However, improvements are needed specifically in tertiary structure prediction tasks. Current trends in PLM development focus on scaling parameters and optimizing training datasets. Nevertheless, indiscriminate model size increases may escalate resource demands and operational costs without addressing all challenges effectively. Future explorations of LMs in the field of proteins not only involve scaling up models but also distinguish PLMs from LMs in NLP. This approach focuses on enhancing models tailored to the characteristics of protein sequences. Additionally, multi-task or multi-modal learning represents promising avenues for further investigation.

## Figures and Tables

**Table 1:** Tools for constructing MSA and protein language model.

Methods	URLs	Classification	Objective
PEbA	https://github.com/mgtools/PEbA	Dynamic programming-based pairwise alignment	MSA for protein monomer
EBA	https://git.scicore.unibas.ch/schwede/EBA		
ClustalW	https://www.genome.jp/tools-bin/clustalw	Multiple sequence alignment	
MAFFT	https://www.ebi.ac.uk/jdispatcher/msa		
MUSCLE	https://www.ebi.ac.uk/jdispatcher/msa/muscle?stype=protein		
T-Coffee	https://www.ebi.ac.uk/jdispatcher/msa		
vcMSA	https://github.com/clairemcwhite/vcmsa		
FASTP	https://fasta.bioch.virginia.edu/fasta_www2/fasta_www.cgi	Sequence-based approaches	
FASTA	https://fasta.bioch.virginia.edu/fasta_www2/fasta_www.cgi		
BLAST	https://blast.ncbi.nlm.nih.gov/Blast.cgi		
Gapped BLAST	https://blast.ncbi.nlm.nih.gov/Blast.cgi		
PSI-BLAST	https://github.com/ianpotpie/psi-blast		
DIAMOND	https://github.com/bbuchfink/diamond		
SAM	http://www.cse.ucsc.edu/research/compbio/	HMM-based approaches	
HMMER	https://github.com/EddyRivasLab/hmmer		
HHsearch	https://github.com/soedinglab/hh-suite		
HHblits	https://github.com/soedinglab/hh-suite		
USEARCH	https://github.com/rcedgar/usearch12	*k*-mer-based approaches	
MMseqs2	https://github.com/soedinglab/MMseqs2/releases		
DeepMSA2	https://zhanggroup.org/DeepMSA/	Multi-stage hybrid approaches	
pLM-BLAST	https://github.com/labstructbioinf/pLM-BLAST	Deep learning-based approaches	
PLMsearch	https://github.com/maovshao/PLMSearch		
DCTdomain	https://github.com/mgtools/DCTdomain		
Evcomplex	http://evcomplex.org/	Genomic distance-based approaches	MSA for protein complex
GremlinComplex	http://gremlin.bakerlab.org/complexes/		
ComplexContact	http://raptorx6.uchicago.edu/ComplexContact/	Phylogeny-based approaches	
cpxDeepMSA	https://zhanggroup.org/cpxDeepMSA/	Protein-protein interactions databases-based approaches	
ESMpair	https://github.com/allanchen95/ESMPair	PLM-based approaches	
DiffPALM	https://github.com/Bitbol-Lab/DiffPALM		
DeepMSA2- Multimer	https://zhanggroup.org/DeepMSA/	Hybrid approaches	
MULTICOM	https://github.com/BioinfoMachineLearning/MULTICOM3		
FASTN	https://fasta.bioch.virginia.edu/fasta_www2/fasta_www.cgi	Sequence-based approaches	MSA for RNA
BLASTn	https://blast.ncbi.nlm.nih.gov/Blast.cgi		
Nhmmer	http://hmmer.org/download.html	HMM-based approaches	
Infernal	https://github.com/EddyRivasLab/infernal	CM-based approaches	
RNAlien	https://github.com/eggzilla/RNAlien	Hybrid approaches	
RNAcmap	https://github.com/jaswindersingh2/RNAcmap		
rMSA	https://github.com/pylelab/rMSA		
MSA transformer	https://github.com/rmrao/msa-transformer	With MSA as input	PLMs
MSA2Prot	/		
ESM-1b	https://github.com/facebookresearch/esm	Autoencoding objectives with single-sequence input	
ProteinBERT	https://github.com/nadavbra/protein_bert		
Saprot	https://github.com/westlake-repl/SaProt		
AminoBERT	https://github.com/zengsihang/AminoBERT-PyTorch		
ESM-2	https://github.com/facebookresearch/esm		
OmegaPLM	https://github.com/HeliXonProtein/OmegaFold		
ProtTrans	https://github.com/agemagician/ProtTrans	Hybrid objectives with single-sequence input	
ProGen	https://github.com/salesforce/progen	Autoregressive objectives with single-sequence input	
ProGen2	https://github.com/enijkamp/progen2		
RITA	https://github.com/lightonai/RITA		
ProtGPT2	https://huggingface.co/docs/transformers/main_classes/trainer		
Tranception	https://github.com/OATML-Markslab/Tranception		
xTrimoPGLM	https://github.com/ONERAI/xTrimoPGLM	Others	

The date of access for all links (accessed on 9 September 2024).

**Table 2:** The advantages and limitations of each type of methods.

Advantages	Limitations	Classification	Objective
Such methods perform well on short sequences or sequences with high similarity.	Such methods have limited sensitivity to distantly related homologous sequences.	Sequence-based approaches	MSA for protein monomer
Such methods can significantly improve sensitivity and alignment quality, allowing for better capture of distant homology.	When the database is very large, the running speed can be slow, especially for complex model training and alignment processes.	HMM-based approaches	
Such methods enable fast and accurate searching of large-scale databases, further enhancing speed and sensitivity.	There is still potential for improving the precision of the MSAs it generates.	*k*-mer-based approaches	
Such methods enable fast and highly sensitive exploration of metagenomic databases, integrating multiple specialized tools to generate optimal MSAs.	The algorithm is complex and requires substantial computational resources.	Multi-stage hybrid approaches	
Such methods significantly improve the sensitivity for identifying homologous query target pairs with low sequence consistency but high structural similarity.	In the local mode, alignments are often shorter yet more accurate, and their evolutionary significance is still to be explored.	Deep learning-based approaches	
The algorithm is simple and intuitive, requiring no additional information.	Such methods are more suitable for prokaryotes.	Genomic distance-based approaches	MSA for protein complex
It addresses the issue that, in eukaryotes, a single MSA containing a rich set of paralogs may pose a challenge for methods based on genomic distance, which are unable to identify potential interactions.	The abundant homologous sequences in metagenomic databases cannot be fully utilized to guide the assembly of multi-chain structures.	Phylogeny-based approaches	
Integrating protein interaction databases for MSA refinement can help produce more stable results	Such MSA construction methods are all hand-crafted approaches and merely have effects on the specific domains.	Protein-protein interactions databases-based approaches	
Such methods enable highly automated MSA concatenation.	The feasibility and effectiveness of its practical application remain to be evaluated.	PLM-based approaches	
Such methods integrate various homologous detection strategies and monomer MSA concatenation techniques to achieve high-quality, deep, and versatile MSA construction.	The construction of MSA for heteromeric complexes requires further improvement.	Hybrid approaches	
Such methods perform well on short sequences or sequences with high similarity.	Such methods have limited sensitivity to distantly related homologous sequences.	Sequence-based approaches	MSA for RNA
HMM-based methods offer enhanced capability for capturing remote homologous relationships compared to sequence-based methods.	These methods lack the utilization of RNA secondary structure information.	HMM-based approaches	
CM-based approaches utilize conserved secondary structure features as supplementary information, which is particularly important for identifying functionally similar RNA molecules with significant sequence divergence.	These methods rely on predefined consensus models, and their performance may be suboptimal when applied to unknown RNA sequences.	CM-based approaches	
These methods integrate various MSA techniques to achieve high-quality, deep, and versatile MSA construction.	The algorithm is complex and requires substantial computational resources.	Hybrid approaches	
Compared to single-sequence input, the results of such methods yield better performance for downstream tasks.	The demand for computational resources is higher.	With MSA as input	PLMs
Implicitly and more effectively capturing the evolutionary and co-evolutionary information of sequences, reducing time costs. The autoencoding-based bidirectional learning is better at learning the contextual relationships of amino acids.	PLM-based methods with autoencoding objectives perform comparably to MSA-based methods in general protein understanding tasks but exhibit relatively lower accuracy in structure prediction.	Autoencoding objectives with single-sequence input	
Autoregressive objectives are more suitable for protein generation tasks	These methods do not adequately capture the complex global interactions of amino acids.	Autoregressive objectives with single-sequence input	
These methods combine the advantages of both autoencoding and autoregressive objectives.	These methods lack design specifically tailored to the features of protein sequences.	Others	

## Data Availability

No new data were created or analyzed in this study. Data sharing is not applicable to this article.

## Abbreviations

MSA, multiple sequence alignment; 3D, three-dimensional; AI, artificial intelligence; PSSMs, position-specific scoring matrices; HMMs, Hidden Markov models; TBM, template-based modeling; GO, Gene Ontology; PTMs, protein post-translational modifications; SVM, support vector machine; PSFMs, position-specific frequency matrices; IDPs, intrinsically disordered proteins; MoRFs, molecular recognition features; PLMs, protein language models; CM, covariance model; NWalign, Needleman-Wunsch algorithm; SWalign, Smith-Waterman algorithm; SP, sum of all pairs; vcMSA, vector-clustering Multiple Sequence Alignment; ML, maximum likelihood; MEGA, Molecular Evolutionary Genetics Analysis; UPGMA, unweighted pair-group method with arithmetic means; BLAST, Basic Local Alignment Search Tool; BLOSUM, Blocks Substitution Matrix; VTML, variable time maximum likelihood; nr, NCBI’s nonredundant Protein Sequence Database; UniProt, Universal Protein Resource; M, match; I, insert; D, delete; HSPs, high-scoring segment pairs; IMG, Integrated Microbial Genomes; pLDDT, Predicted Local Distance Difference Test; Nf, number of effective sequences; NT, NCBI nucleotide sequence database; CASP, Critical Assessment of protein Structure Prediction; NLP, Natural Language Processing; ENA, genome database; PDB, Protein Data Bank; MLM, masked language modeling; rSS, RNA secondary structures; SCFGs, Stochastic context-free grammars; QDB, query-dependent banded; 3Di, 3D interaction; RoPE, Rotary Position Embedding; BPE, Byte Pair Encoding; GLM, General Language Model; PPI, protein–protein interaction; DCT, discrete cosine transformation.
